# Plasma Dilution After Myocardial Ischemia–Reperfusion Injury Promotes Cardiac Repair, Heart Performance, and Recovery of Motor Function and Endurance in Old Mice

**DOI:** 10.1111/acel.70525

**Published:** 2026-04-30

**Authors:** Joana Marie C. Cruz, Rana Alzalzalee, Hayden Yeung, Zainab Mahmood, Qile Yang, Negar Morshedian, Zachery R. Robinson, Karan R. Malhotra, Michael J. Conboy, Ahmad Reza Mazahery, Jose B. Nevado, Irina M. Conboy

**Affiliations:** ^1^ Department of Bioengineering and QB3 Institute University of California Berkeley Berkeley California USA; ^2^ College of Medicine, University of the Philippines Manila Manila Philippines; ^3^ Institute of Biology, College of Science University of the Philippines Quezon City Philippines; ^4^ National Institutes of Health (NIH), University of the Philippines Manila Manila Philippines; ^5^ Generation Lab, Inc. Burlingame California USA

**Keywords:** aging, blood, blood exchange, cardiovascular diseases, myocardial ischemia–reperfusion, plasma, plasma dilution

## Abstract

Myocardial infarction (MI) is the leading cause of cardiovascular‐related deaths worldwide, with risk increasing sharply with age. Fibrosis and inflammation occur soon after a pathological event and reflect perturbation of tissue repair that accompanies aging in general. Yet not old, but young animals are typically used for studying MI, emphasizing the unmet need for more relevant preclinical models. We previously determined that plasma dilution, also termed neutral blood exchange (NBE) (replacing ~50% of plasma with saline containing 5% albumin) broadly promotes tissue repair and maintenance, reduces fibrosis and inflammation in old mice, and improves the health of humoral and cellular compartments of blood in old people. Here, we developed a novel preclinical model via combining two complex surgeries in aged mice: jugular vein cannulation followed by plasma dilution with open heart cardiac ischemia–reperfusion (I/R). Using this approach, we found that a single dilution of old plasma that was performed 24 h after I/R significantly and broadly improved the recovery at molecular, cellular, tissue, and functional levels in aged mice. Candidate molecular pathways (JAK/STAT and TGF‐β) and target proteins associated with these beneficial effects have been suggested by the bioinformatics on comparative proteomics between sham, I/R, and I/R plus NBE groups. While future studies are needed to establish the detailed processes and safety profiles of plasma dilution as a treatment of MI, this work provides important translational and mechanistic insights into recovery from cardiac injury in aged patients.

## Graphical abstract

Plasma dilution at 24 h after I/R improved cardiac remodeling, function, and the physical performance of old male and female mice, through lowered markers of inflammation, fibrosis, reduction of cardiomyocytes' apoptosis, increase in their cell‐cycle re‐entry, and healthier profile of newly synthesized proteome, including the determinants of Jak/Stat pathway.

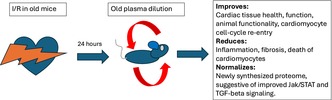

## 1 Introduction

Myocardial infarction remains the leading cause of disability and mortality worldwide, with predisposition to the disease increasing significantly after the age of 65 (Engberding and Wenger 2017). Despite significant advancements in mitigating the complications of MI, problems with subsequent decline in physical performance, cardiac complications, and heart failure (HF) remain. 11%–14% of patients are readmitted 30 days post‐MI due to angina, heart failure, and non‐cardiovascular complications (e.g., kidney failure, respiratory distress, stroke) (Wang et al. 2019; Vallabhajosyula et al. 2019) and 43% of MI patients experience decline in physical function and independence (Dodson et al. 2012). Therefore, there is a need to develop better treatment strategies for ameliorating the decline in cardiac function and long‐term complications throughout the body, particularly, in elderly patients.

Myocardial ischemia–reperfusion (I/R) injury is a harmful cardiovascular consequence that occurs after the restoration of blood flow following arterial occlusion. It commonly arises in patients undergoing percutaneous coronary intervention (PCI) or receiving thrombolytic therapy (Zhang, Liu, et al. 2024; Hausenloy and Yellon 2013). Moreover, while restoring blood flow to the ischemic area is necessary, it paradoxically leads to further tissue damage due to an influx of calcium and an increase in reactive oxygen species (ROS) (Zhang, Jiang, et al. 2024). With aging, ongoing high levels of oxidative stress and inflammation make arteries more vulnerable to I/R (Bencurova et al. 2023; Heusch 2024). In old mice, 30 min blood flow deprivation leads to more severe consequences than in young, including pathological remodeling of cardiac tissue, impaired cardiac function, and reduced overall physiological performance (Przyklenk et al. 2008; Willems et al. 2005). These outcomes highlight the age‐related decline in tissue maintenance and repair after MI or I/R—a phenomenon observed in both mice and humans. Because the underlying mechanisms remain only partially understood, developing reliable pre‐clinical models of myocardial injury in older subjects is both challenging and important.

Following MI or I/R, a multitude of systemic changes are triggered, including inflammation, metabolic disturbances, and neurohormal alterations (Fang et al. 2015; Kohsaka et al. 2005; Moon et al. 2019; Peng et al. 2024). On one hand, these processes overlap with and promote tissue repair; however, when excessively activated, they drive maladaptive cardiac remodeling and contribute to multi‐organ complications. Interestingly, humoral and cellular compartments of blood (the systemic milieu) play central roles in regulating these responses, modulating inflammation, fibrosis, oxidative stress, and endothelial function/dysfunction (Węgiel and Rakowski 2021; Chan et al. 2020; Feng et al. 2023). Some of the key circulating factors that participate in post‐MI responses include cytokines (e.g., TNF‐α, IL‐6), growth factors (e.g., PDGF, VEGF), and antibodies.

Despite the recognized importance of systemic changes in MI and I/R pathophysiology, effective therapeutic strategies and comprehensive preclinical and clinical studies on their alterations remain limited. Current systemic approaches are largely restricted to the removal of C‐reactive protein (CRP) via apheresis or infusion of autologous plasma components (Ries et al. 2018, 2021), highlighting the need to investigate MI‐related therapeutic approaches of deliberately modifying the systemic milieu, such as through therapeutic plasma exchange (TPE). This procedure is widely used to remove pathogenic substances, including autoantibodies, immune complexes, and toxins, and is commonly employed to treat autoimmune and antibody‐mediated conditions (Moriguchi et al. 2017; Koizumi et al. 2017). TPE is currently being applied to treat atherosclerosis, myocarditis, cardiomyopathy, and autoimmune or metabolic‐related cardiac dysfunctions (Koizumi et al. 2017; Moriguchi et al. 2017; Kulikova et al. 2020; Marlęga‐Linert et al. 2024). Its therapeutic potential for cardiac injuries has not been comprehensively explored. A small animal model of TPE, which is called neutral age blood exchange (NBE), demonstrated that dilution of old blood plasma with saline that contains albumin (replenishing this procedure‐removed systemic protein) reduces fibrosis of muscle and liver and restores the levels of systemic proteins to their youthful healthier profile (Mehdipour et al. 2020, 2022).

To improve the understanding of the effects of systemic milieu on recovery from cardiac injury, and moreover, to apply such understanding to the very relevant aged group, we developed a preclinical model of NBE in aged male and female mice that underwent experimental myocardial I/R injury and used it for uncovering a novel therapeutic capacity of plasma dilution to treat MI. Specifically, our data demonstrate that a single NBE one day after cardiac I/R significantly improved cardiac remodeling, preserved cardiac function, and enhanced the physical performance of old animals. Furthermore, NBE administered to old mice at 24 h post‐MI significantly lowered inflammation, fibrosis, and apoptosis at 5 days following cardiac injury. Based on the bioinformatics analyses of de‐novo, metabolically tagged proteomes, several JAK/STAT signaling determinants, as well as total levels of TGF‐β pathway proteins important for cardiac health, were involved with the effects of NBE. Although the clinical applicability of plasma dilution after MI remains to be fully defined, this study provides evidence on expanding the critical therapeutic window to 24 h after the event.

## 2 Results

### 2.1 NBE Improves Cardiac Function and Physical Performance of Old Animals After Ischemia–Reperfusion

To determine the effects of NBE on disease pathophysiology after MI, we first developed a model of myocardial infarction in 18‐ to 22‐month‐old male and female C57BL/6 mice. I/R injury with 40‐ or 60‐min ischemia resulted in markedly increased mortality of the old animals, while 30‐min I/R was well tolerated (Figure S1). Hence, 30‐min I/R was applied in the subsequent experiments.

**FIGURE 1:**
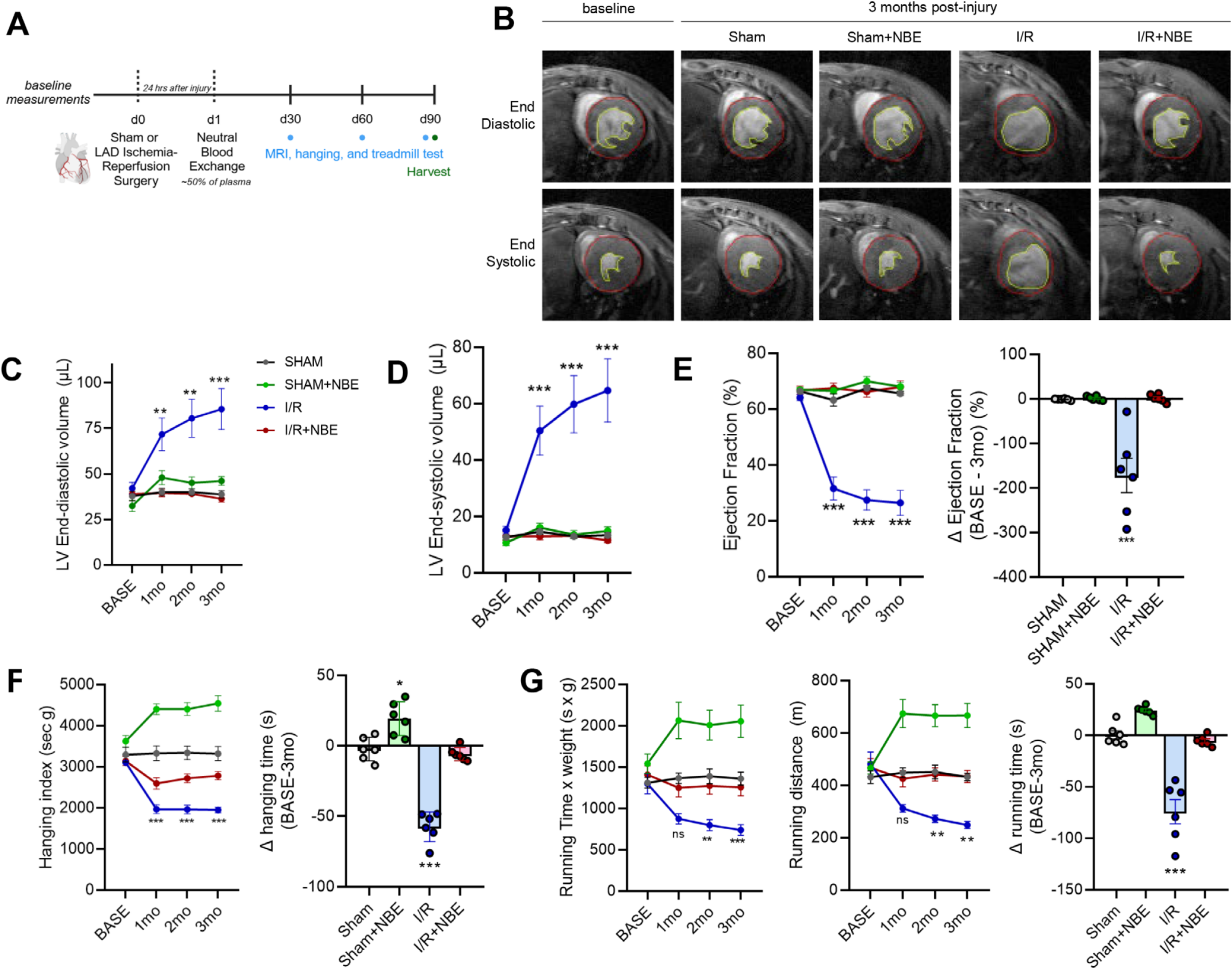
NBE improves cardiac function and physical performance after ischemia–reperfusion. (A) Schematic of the study. NBE was performed 24 h after 30 min of myocardial ischemia–reperfusion (I/R) injury. Cardiac function and physical performance were assessed every month for 3 months. (B) Transversal short‐axis MRI images at end‐diastolic (ED) and end‐systolic (ES) 3 months after surgery showed ventricular dilatation. (C–E) Cine‐MRI measurements of cardiac morphology and function every month for 3 months post‐I/R revealed preserved EDV, ESV, and ejection fraction (EF) in I/R + NBE group. (F, G) Hanging indices and running distance over time for 3 months and percent (%) change from baseline showed preserved motor function and endurance in I/*R* + NBE group. Data are shown as the mean ± SE (*n* = 6). Sham versus I/R *p < 0.05, **p < 0.01, and ***p < 0.001.

Considering that immediate intervention is the standard of care post MI (Rao et al. 2025), we also decided to test if NBE has the capacity to broaden this therapeutic window; we administered NBE 24 h post I/R: after the onset of cardiac tissue death, but before the peak of injury response that takes place ~72–96 h after the event (Prabhu and Frangogiannis 2016) (Figure 1A).

Sequential cardiac magnetic resonance imaging (MRI) was performed to assay cardiac function, comparing the sham procedure (open heart surgery, but no ischemia) with I/R (alone or plus NBE), as well as NBE alone. Left ventricular (LV) end‐diastolic volume (EDV), LV end‐systolic volume (ESV), and ejection fraction (EF) were measured. Consistent with published data, I/R resulted in LV dilation and cardiac dysfunction exhibited by increased LV EDV and ESV, and reduced ejection fraction (EF) (Figure 1B–E). This change was most notable at 1‐month post‐I/R with gradual increase in LV EDV and ESV and decrease in EF over the post‐I/R time course. In contrast, the I/*R* + NBE group had much better‐preserved cardiac parameters, which were statistically indistinguishable from the sham group. NBE alone did not induce noticeable changes in cardiac function, as compared to the sham (Figure 1).

As cardiac function significantly influences physical performance (Landi et al. 2018), we tested the motor function and endurance of the mice through four‐limb hanging test and treadmill exhaustion test, respectively. Consistent with the parameters of heart health, hanging time and running distance were diminished at 1‐month post‐I/R and declined by more than 50% from the baseline measures at 3 months after I/R. Importantly, functional performance was preserved in the I/R + NBE mice, reaching levels comparable to those of the sham group (Figure 1F,G).

It is noteworthy that even the old sham + NBE mice had improved hanging indices and treadmill running capacity as compared to the sham alone group, supporting and expanding the paradigm of broad rejuvenation through plasma dilution (Mehdipour et al. 2020).

We also investigated liver health, given its central role in systemic metabolism, inflammation, post‐I/R recovery (Bannon et al. 2021; Samsky et al. 2013; Lonardo et al. 2024; Fabbrini and Magkos 2015) and because the liver is the primary source of plasma albumin, which is removed and replaced during NBE (Baral 2025). Using Masson's trichrome and Oil Red O assays, we found no significant increase in liver adiposity or fibrosis from NBE, supporting the therapeutic feasibility of this procedure (Figure S2A–D). Liver proteomics that was done at 3 months post I/R showed that IL‐23 was mildly upregulated in the I/*R* + NBE group, as compared to the I/R alone: of note, this general inflammatory protein (García‐Domínguez 2025) is, interestingly, critically needed for cardiac recovery and wound healing after I/R (Savvatis et al. 2014) (Figure S2E). The approaches that were utilized in the liver studies are detailed in Figures S3 and S4.

These results suggest that NBE at 24 h post I/R confers multiple protective health benefits to old animals. It preserves cardiac structure and function (LV EDV, LV ESV, and EF), maintains physical performance and endurance, and does so without inducing hepatic fibrosis or lipid accumulation. No differences in the above outcomes were observed between males and females (Figure S5).

### 2.2 NBE Promotes Healthy Cardiac Remodeling After Ischemia–Reperfusion

**FIGURE 2:**
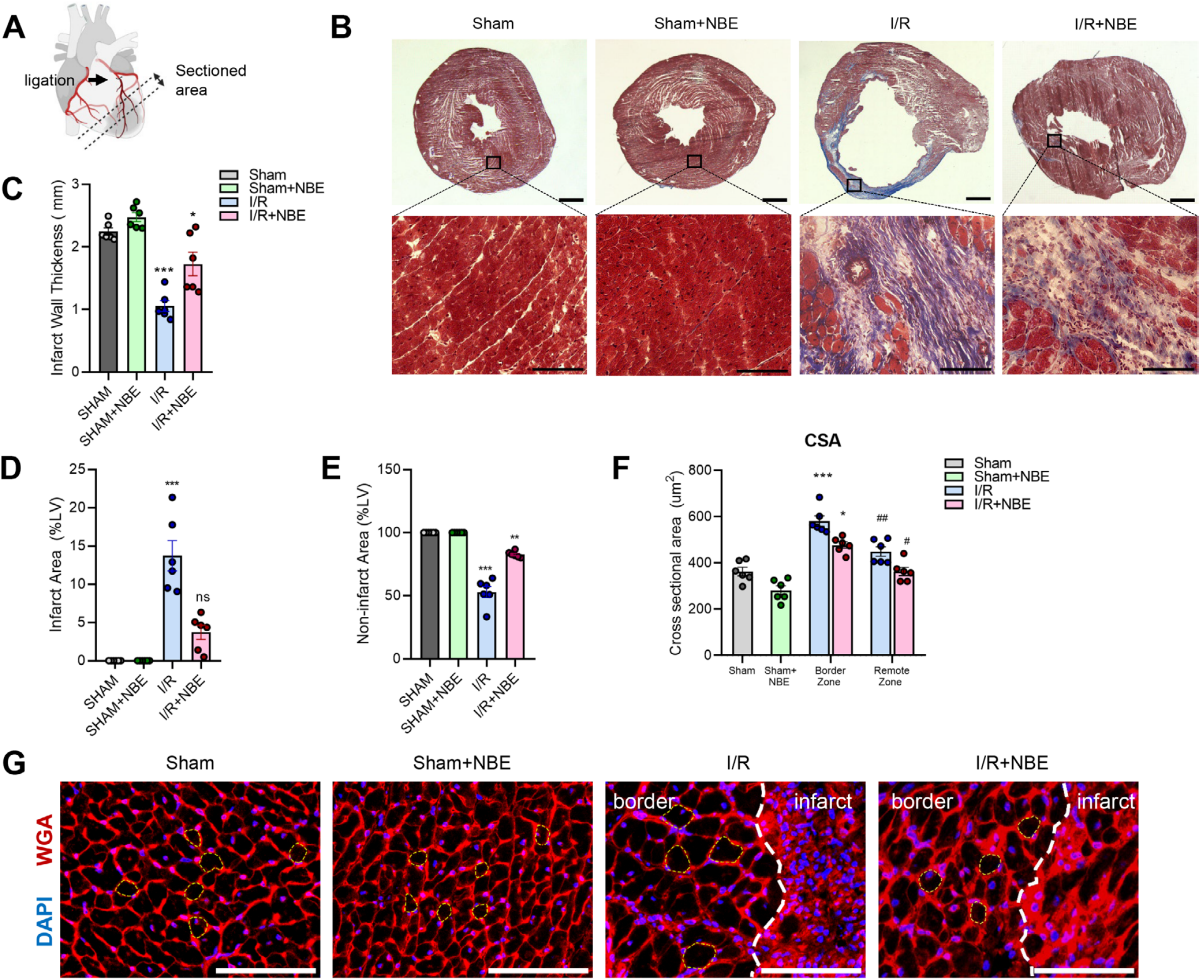
NBE alleviates cardiac remodeling after ischemia–reperfusion. (A) Left anterior descending (LAD) artery was ligated and sections were cut below the ligation point and a third from the apex. (B) Representative Masson's' trichrome images 3 months after injury per treatment showed reduced collagen deposition in I/R + NBE group. Scale: 1 mm for the full heart sections and 100 μm for the close‐up images. (C) Wall thickness (in mm) in the infarct area 3 months after injury were preserved in I/R + NBE mice. (D, E) Percent infarct area and viable area. (F, G) Quantification and representative images of WGA staining for cardiomyocyte area revealed no significant changes on hypertrophy in I/R + NBE group. All counts were made in border zone and remote zone. Scale: 100 μm. Data are shown as the mean ± SE (*n* = 6). *p < 0.05, **p < 0.01, and ***p < 0.001 for sham versus I/R and #*p* < 0.05, ##*p* < 0.01, and ###*p* < 0.001 for I/R + NBE versus I/R.

To link the functional improvements after NBE in old mice to cardiac tissue health, we next assessed myocardial fibrosis and hypertrophy which are key pathological changes typically occurring several weeks post‐injury (Sanjaya 2025; Rusu et al. 2019). Accordingly, we examined these structural changes 3 months after I/R in the male and female mice. Cryosectioning at 10‐μm was performed throughout the heart, and sections were analyzed which were obtained below the ligation point and at least a third from the apex (Figure 2A).

For assessing fibrosis, we applied Masson's trichrome staining and quantified the fibrotic and unaffected areas. Demonstrating significantly better repair at injury sites, the I/R + NBE group had reduced fibrosis across the endocardial, myocardial, and epicardial layers, whereas the I/R group exhibited transmural fibrosis (Figure 2B).

We also evaluated the thickness of infarct wall, the infarct area, and non‐infarct area. Based on each of these parameters, NBE promoted healthier cardiac remodeling after I/R in the old mice: the I/R + NBE mice had thicker infarct wall, smaller infarct area, and larger viable myocardium (Figure 2C–E).

Next, we used wheat germ agglutinin (WGA) staining to evaluate the cardiac hypertrophy, which established that cardiomyocyte sizes surrounding the infarct area of I/R + NBE group were notably smaller than those of I/R alone (Figure 2F,G). As above, there were no sex‐specific differences for these parameters in the studied cohorts (Figure S6).

Interestingly and consistently with the above described improvements in the performance of old mice, NBE reduced heart hypertrophy even in the aged sham mice, as compared to sham without NBE, confirming the rejuvenation capacity of old plasma dilution (Mehdipour et al. 2020) and extending it to the reversal of this hallmark of heart aging (Abdellatif et al. 2023).

### 2.3 NBE Reduces Inflammation and Fibrosis During Remodeling of Aged Cardiac Tissue

Cardiac remodeling after I/R is initiated by several processes, including cardiomyocyte death, inflammation, fibrosis, and apoptosis (Zhang et al. 2022). All these processes are activated during 0–7 days after I/R (Zhang et al. 2022), with the inflammatory phase peaking at approximately 72–96 h and beginning to resolve by Day 7, transitioning into a reparative phase (Prabhu and Frangogiannis 2016; Ong et al. 2018; Halade and Lee 2022). Considering these timelines, we analyzed the markers of cardiac inflammation, fibrosis, apoptosis, and senescence at 5 days post I/R: a critical time window for resolving inflammation and the onset of fibrotic remodeling.

**FIGURE 3:**
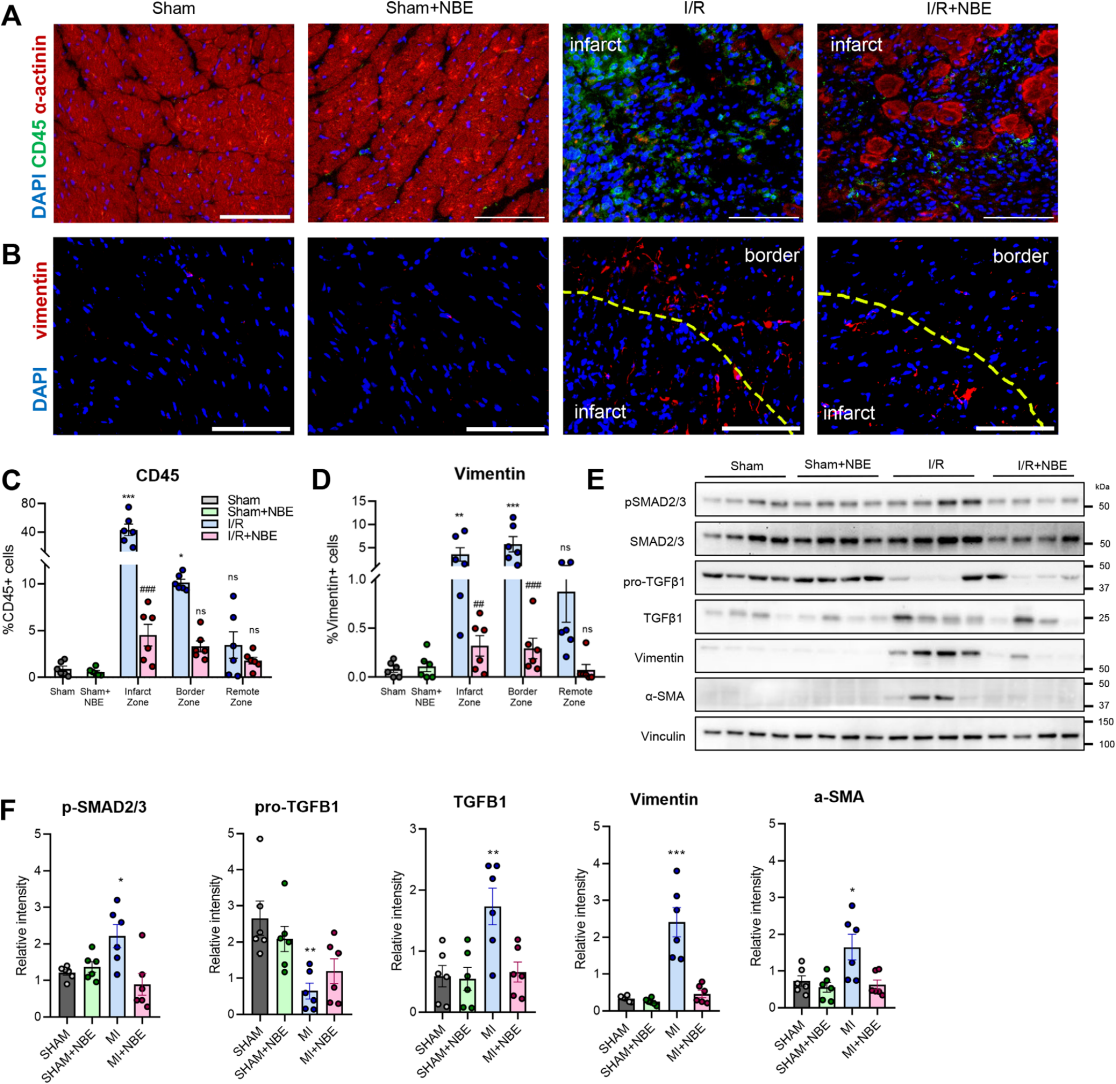
NBE reduces inflammation and fibrosis after ischemia–reperfusion. (A) Representative CD45 immunofluorescent images obtained from all groups at day 5 after injury or sham group showed reduced infiltration of CD45+ cells in I/R + NBE group. Shown are CD45 (green), alpha actinin (red), and DAPI (blue). Scale: 100 μm. (B) Representative vimentin immunofluorescent images obtained from all groups at Day 5 after injury or sham operation showed reduced cardiac fibroblasts around the infarct and border area in I/R + NBE mice. Shown are vimentin (red) and DAPI (blue). Scale: 100 μm. (C) Percentage of CD45‐positive cells in the left ventricle of samples across all groups. (D) Percentage of Vimentin‐positive cells in the left ventricle of samples across all groups. All counts were made in infarct zone, border zone, and remote zone. (E) Representative immunoblots detecting fibrosis markers such as pSMAD2/3, SMAD2/3, pro‐TGFβ1, TGFβ1, Vimentin, α‐smooth muscle Actin (SMA), and vinculin confirmed reduced fibrosis in I/R + NBE group. (F) Western blot analysis. Data are shown as the mean ± SE (*n* = 6). *p < 0.05, **p < 0.01, and ***p < 0.001 sham versus I/R and #*p* < 0.05, ##*p* < 0.01, and ###*p* < 0.001 I/R + NBE versus I/R.

Immune cells in cardiac tissue were identified by immunofluorescence for the pan‐leukocyte marker, CD45, which is widely used for assaying tissue inflammation (Wu et al. 2023; White et al. 2014). The cardiac marker alpha‐actinin was used for co‐immunodetection and 10‐μm cryosections throughout the infarcted area of the hearts were analyzed. The results demonstrated that, as compared to I/R alone, I/R + NBE mice exhibited a significant reduction in CD45^+^ infiltrating leukocytes within the infarcted areas. Corroborating this approach, in remote I/R zones, low numbers of CD45^+^ cells were observed across all cohorts, as expected (Figure 3A,C).

Following the early inflammatory phase, myocardial fibrosis deposits collagen and produces scar tissue (Frangogiannis 2021; Francis Stuart et al. 2016). To assess this across the cohorts, the cardiac fibroblast marker vimentin was detected by immunofluorescence (Doppler et al. 2017). The results revealed elevated numbers of vimentin‐expressing cells in the infarct and border zones of I/R hearts, whereas I/*R* + NBE mice exhibited minimal levels (Figure 3B,D). Figure S7 shows no differences between male and female mice in these parameters.

Consistent with these observations, pro‐fibrotic markers, such as, phosphorylated SMAD2/3, TGFβ1, vimentin, and α‐smooth muscle actin (SMA), were significantly lower in the I/*R* + NBE group as compared to the I/R alone in Western blotting (Figure 3E,F). Importantly, NBE alone did not induce infiltration of inflammatory cells, activate cardiac fibroblasts, or upregulate the tested fibrotic markers, in support of its clinical usability.

Together, these findings suggest that NBE ameliorates multiple features of cardiac inflammation and fibrosis following ischemia–reperfusion.

### 2.4 NBE Attenuates Apoptosis and Senescence of Cardiomyocytes After Ischemia–Reperfusion

Ischemia‐induced apoptosis and senescence are key contributors to cardiomyocyte loss after I/R, which aggravate myocardial fibrosis (Bu et al. 2024; Yan et al. 2021; Redgrave et al. 2023). Thus, to further examine the mechanisms by which NBE promotes recovery of old mice from I/R, we performed terminal deoxynucleotidyl transferase–mediated nick‐end labeling (TUNEL) with anti‐alpha actinin co‐immunodetection, in the 10‐μm heart cryosections at 5 days after MI.

**FIGURE 4:**
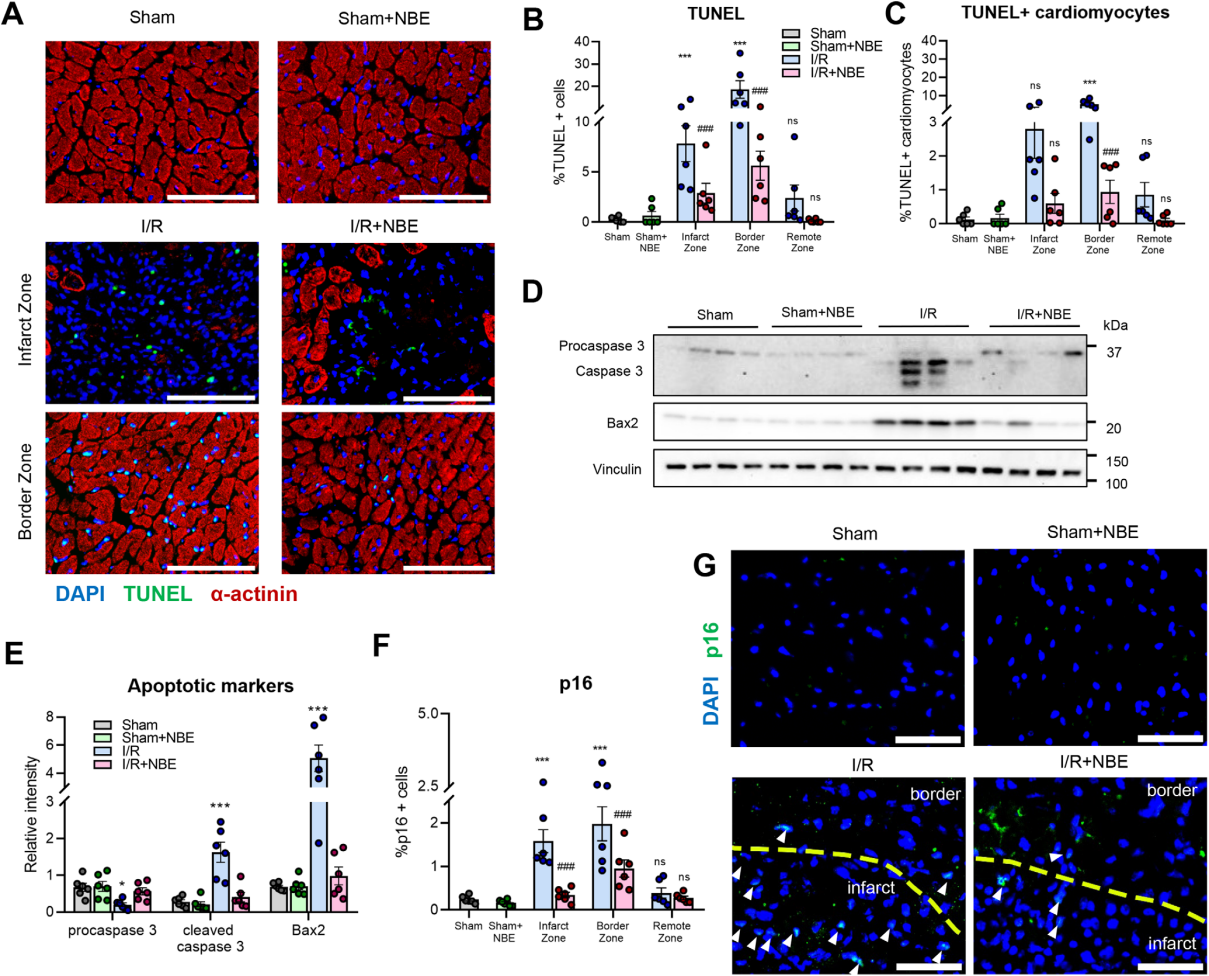
NBE attenuates apoptosis and senescence after ischemia–reperfusion. (A) Representative TUNEL immunofluorescent images obtained from all groups at day 5 after injury or sham operation revealed reduced apoptotic cells in I/*R* + NBE group. Shown are TUNEL (green), alpha actinin (red), and DAPI (blue). scale: 100 μm. Percentage of (B) TUNEL‐positive cells and (C) TUNEL+ cardiomyocytes in the left ventricle of samples across all groups. All counts were made in infarct zone, border zone, and remote zone. (D) Representative immunoblots detecting apoptotic markers such as procaspase 3, cleaved caspase 3, and Bax2 showed reduced expression in I/*R* + NBE group. (E) Western blot analysis. (F, G) Quantification and Representative p16 immunofluorescent images obtained from all groups at Day 5 after injury or sham operation revealed reduced apoptotic cells in I/*R* + NBE group. Data are shown as the mean ± SE (*n* = 6). *p < 0.05, **p < 0.01, and ***p < 0.001 for sham versus I/R and #*p* < 0.05, ##*p* < 0.01, and ###*p* < 0.001 for I/R + NBE versus I/R.

As shown in Figure 4A–C, I/R + NBE mice exhibited significantly fewer apoptotic cardiomyocytes than I/R alone. Corroborating the validity of this approach, TUNEL^+^ cells were localized primarily in the infarct and border regions, as expected. These findings were confirmed by Western blotting studies, which demonstrated downregulation of proapoptotic proteins (caspase‐3 and Bax2) in I/R + NBE mice, as compared to I/R alone, in agreement with the TUNEL data (Figure 4D,E).

Next, we analyzed the in vivo cellular senescence using p16 immunofluorescence. The data demonstrated that p16 positive cells in the hearts were mostly observed near the border of the infarct and that NBE significantly reduced their numbers (Figure 4F,G). The effects of NBE on reducing apoptosis and senescence were similar between male and female mice (Figure S8A–D).

These data establish that NBE influences the key cellular processes, which are associated with the recovery from MI. Namely, a single NBE procedure at 24 h after a 30‐min cardiac ischemia in old mice was sufficient for significantly diminishing apoptosis and senescence of cardiomyocytes.

### 2.5 NBE Promotes Cardiomyocyte Cell Cycle Re‐Entry After Ischemia–Reperfusion

In response to I/R, adequate cardiomyocyte repair, particularly in the border zone, is vital in limiting pathologic cardiac remodeling (Li et al. 2021; Constanty et al. 2025).

Hence, we investigated the impact of NBE on cardiac reparative potential by performing immunofluorescence for Ki67, a marker that persists for up to 24 h of cell division, with co‐immunodetection of cardiac troponin (cTn), which is highly expressed in cardiomyocytes (Liu et al. 2024; Uxa et al. 2021). These analyses were applied to the heart cryosections that were collected at 5 days post I/R with or without NBE, sham, or NBE alone.

**FIGURE 5:**
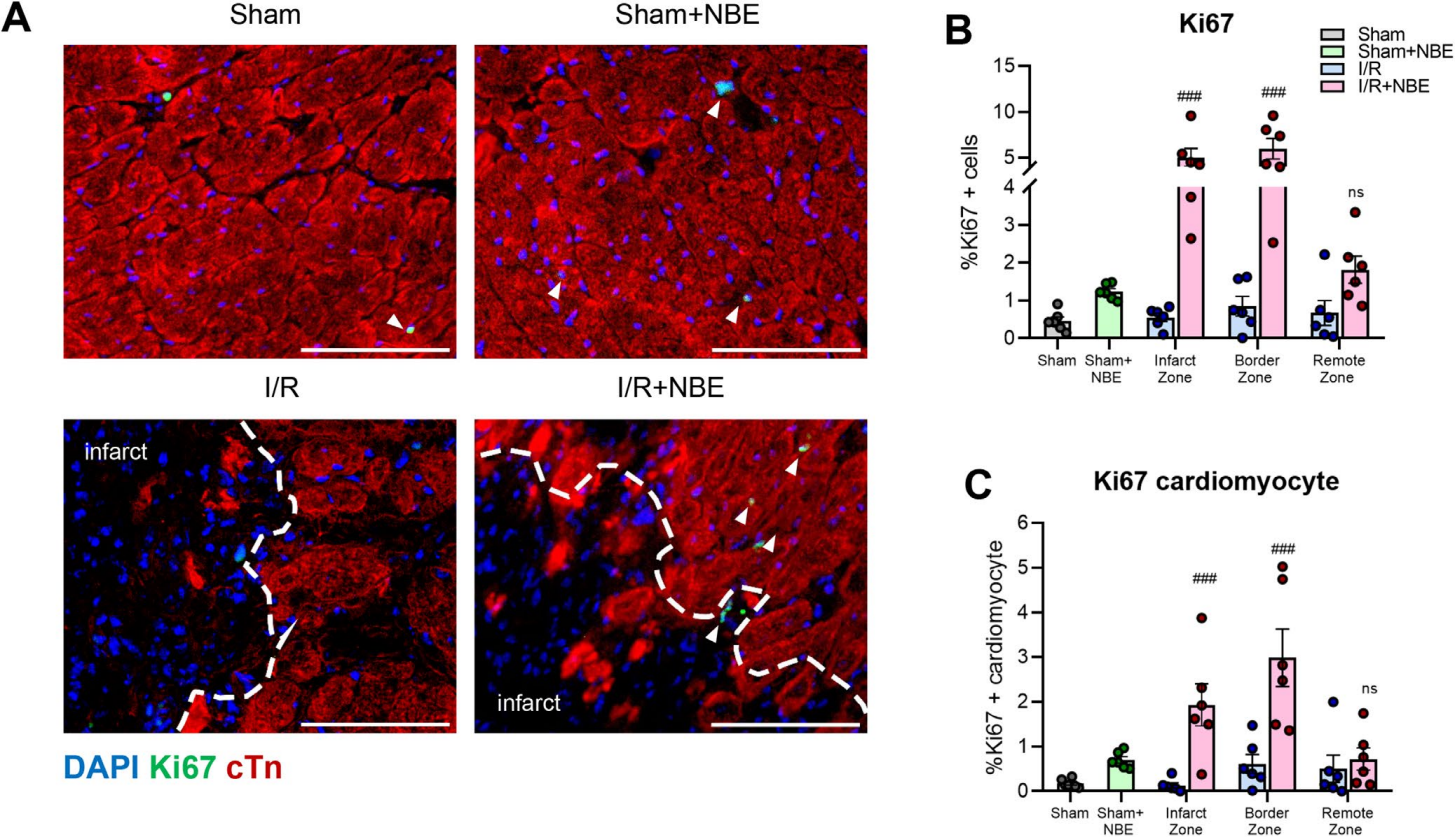
NBE promotes cardiomyocyte cell cycle re‐entry after ischemia–reperfusion. (A) Representative Ki67 immunofluorescent images obtained from all groups at Day 5 after injury or sham operation showed more Ki67+ cells in I/R + NBE group. Shown are Ki67 (green), cardiac troponin7 (red), and DAPI (blue). scale: 100 μm. Percentage of (B) Ki67‐positive cells and (C) Ki67‐positive cardiomyocytes in the left ventricle of samples across all groups. All counts were made in infarct zone, border zone, and remote zone. Data are shown as the mean ± SE (*n* = 6). *p < 0.05, **p < 0.01, and ***p < 0.001 for sham versus I/R and #*p* < 0.05, ##*p* < 0.01, and ###*p* < 0.001 for I/R + NBE versus I/R.

The results of these assays demonstrated that NBE administered to old mice 24 h after I/R significantly increased the numbers of Ki67^+^ cTn^+^ cardiomyocytes, as well as Ki67^+^ cTn^−^ cardiac resident cells in the infarct and border regions compared to I/R alone (Figure 5A–C). This observed increased cell cycle activation of cardiomyocytes and cardiac resident cells was similar for male and female mice (Figure S8E,F).

The enhanced cell cycle re‐entry supports the above‐shown findings on the minimal change in LV wall thickness observed in the I/R + NBE group despite reduced cardiomyocyte size, suggesting that preserved LV thickness may result from cell expansion in addition to reduced cardiomyocyte apoptosis.

These results strengthen the cellular mechanisms by which NBE confers protective benefits to the hearts of old animals after ischemia–reperfusion.

### 2.6 NBE Restores Healthy Cardiac Protein Synthesis at 5 Days Post I/R

**FIGURE 6:**
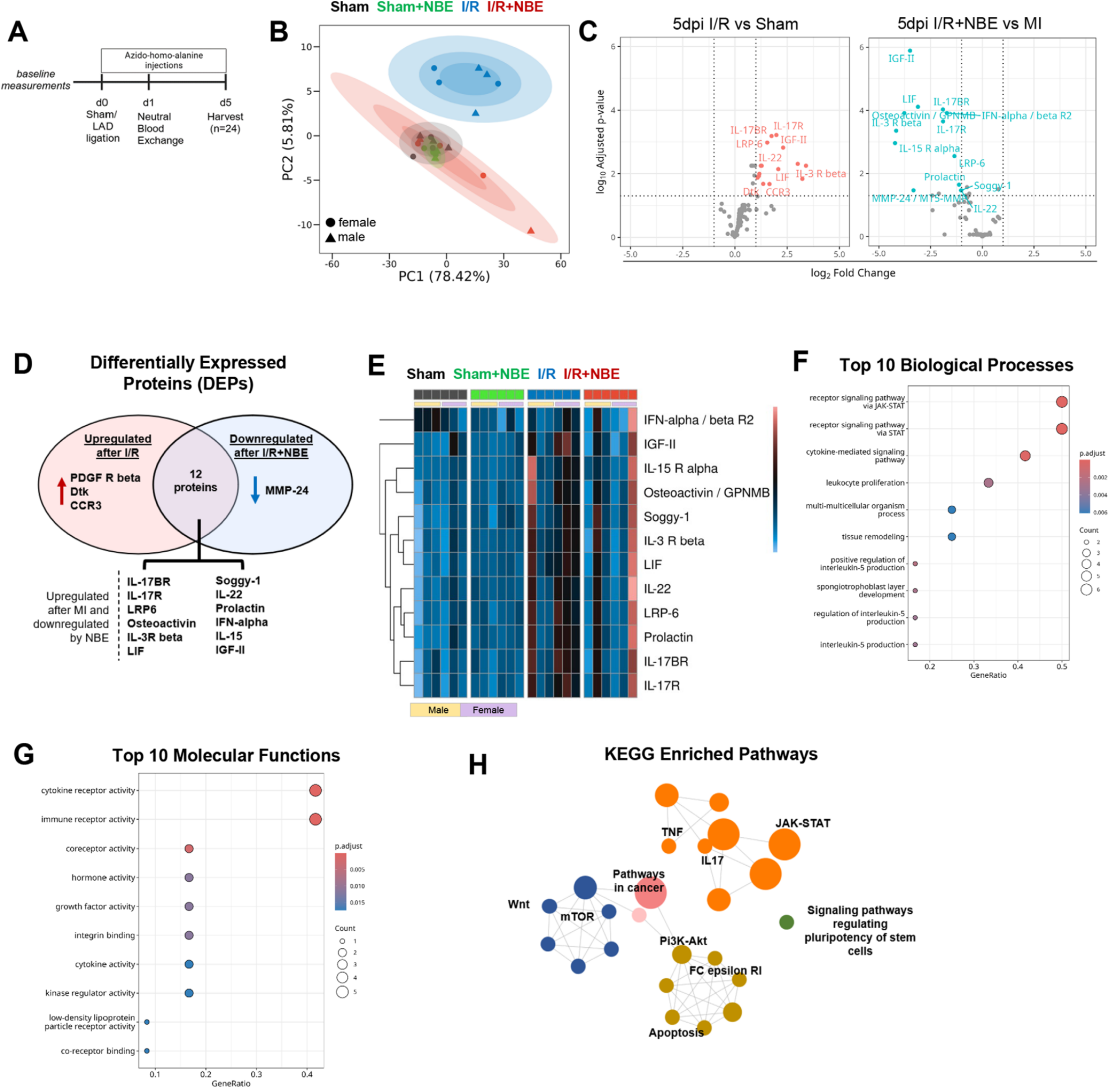
NBE restores de novo cardiac proteome levels to normal. (A) Schematic diagram. NBE was performed 24 h post‐injury. Mice were injected with azido‐homo‐alanine for 5 days starting with the onset of injury. Heart samples were harvested at 5 days post injury. (B) Principal component analysis (PCA) plots of the protein expression profile of all treatment groups showed clear separation of sham and I/R controls, with clustering of I/R + NBE group in sham. Confidence ellipses at 50%, 80%, and 95% intervals are overlaid to indicate group variance. (C) Volcano plots identified differentially expressed proteins (DEPs) in I/R versus Sham controls and I/R + NBE versus I/R controls. Proteins highlighted in red are significantly upregulated while proteins highlighted in blue are significantly downregulated (adjusted *p*‐value < 0.05) (horizontal dotted line), and log2 fold change > 2 (vertical dotted line). (D) Venn diagram showed the 12 overlapping DEPs between the upregulated proteins after injury and their downregulation after NBE treatment. (E) Heatmap visualized the expression of the 12 overlapping proteins across all groups. (F, G) Gene Ontology overrepresentation analysis of the 12 overlapping DEPs highlighted top 10 biological processes and molecular function, respectively. (H) cirFunMap visualization of KEGG enriched pathways of the 12 overlapping proteins.

To address the molecular mechanisms behind the multiple aspects of NBE‐promoted cardio‐protection post I/R, we performed metabolic proteomics, comparing the de‐novo proteins synthesized by heart tissue between the cohorts. Specifically, I/R (alone or with NBE at 24 h post I/R), sham and NBE procedures were followed by 5 daily sub‐cutaneous in vivo administrations of azido‐homo‐alanine (AHA), a non‐canonical amino acid tag. Heart tissues were collected postmortem after the 5 days of AHA and analyzed for the levels of newly synthesized proteins via noncanonical amino acid tagging, as in (Liu et al. 2017, 2022) (Figure 6A).

Principal component analysis (PCA) with sex‐annotated data demonstrated a clear separation of clustering between I/R and sham controls (Figure 6B). Notably, the I/R + NBE group clustered close to the sham and separately from the I/R alone group, suggesting that NBE broadly restored the newly synthesized proteome of cardiac tissue, making it akin to that of sham, in both male and female mice.

To understand the mechanisms by which NBE confers cardio‐protection post‐injury, we identified the differentially expressed proteins (*DEPs: p* < 0.05, fold change > 2) in the sham controls vs. the I/R and in the I/R versus the I/R + NBE groups (Figure 6C). I/R and I/R + NBE mice displayed high heterogeneity in de‐novo protein synthesis, in agreement with increased biological noise during pathologies in the old (Bahar et al. 2006; Ilan 2025; Hernando‐Herraez et al. 2019). No proteins were upregulated in their de‐novo synthesis in the I/R + NBE group as compared to I/R alone (Figure 6C). Interestingly, we found 15 proteins that were significantly elevated in the I/R group, 12 of which were downregulated in the I/*R* + NBE group: IL‐3R beta, IL‐15, IL‐17BR, IL‐17R, IL‐22, LRP6, osteoactivin, leukemia inhibitory factor (LIF), soggy‐1, prolactin, interferon alpha, and insulin‐like growth factors (IGF)‐II (Figure 6D,E).

Bioinformatic analyses demonstrated that I/R‐elevated and I/R‐NBE‐diminished DEPs were highly enriched for heart‐repair biological processes and functions, such as JAK–STAT pathway, leukocyte proliferation, tissue remodeling, IL‐5 production, cytokine‐mediated signaling; activities of cytokines, immune receptors, hormones, growth factors, and low‐density lipoprotein (LDL) receptor (Figure 6F,G).

STRING protein interactome analyses confirmed and expanded the bioinformatics to the main signaling hubs involved in the NBE responsiveness to I/R: de‐novo synthesized determinants of JAK/STAT, TNF, IL‐17, and Pi3/Akt (Figure 6H).

In agreement with the resolution of acute injury and transition to a chronic remodeling (Leancă et al. 2022; Arai 2015), at 3 months post‐I/R, PCA on total heart protein levels showed substantial overlap among all cohorts. The six upregulated proteins—erythropoietin (EPO), frizzled‐1, eotaxin, growth hormone R, hepatocyte growth factor (HGF), and growth differentiation factors (GDF)‐9—were enriched in pathways related to apoptotic regulation, tissue morphogenesis, cytokine/growth factor signaling and converse on IL‐17, Ras1, Rap1, and MAPK networks (Figure S9). These bioinformatics findings are consistent with the key molecular determinants of chronic myocardial remodeling (Akasaka et al. 2006; Ushakov et al. 2020). The full proteomics data are provided in Data S1.

Collectively, these results establish that NBE restores the newly synthesized heart tissue proteome at 5 days post‐injury to a healthier state akin to that of the sham, in part by attenuating de‐novo levels of several JAK–STAT signaling determinants, inflammatory cytokines, and pro‐fibrotic factors.

## 3 Discussion

This study developed a pre‐clinical model of MI with NBE and uncovered the therapeutic potential of plasma dilution for robustly improving the recovery from age‐associated cardiac injury in animals that are equivalent to 60^+^‐year‐old patients. Mice underwent a 30‐min ischemic period followed by recovery, and the treatment group received NBE 24 h later. Our data established that a single NBE procedure administered at this timepoint significantly attenuated the extent of infarction and preserved cardiac function, as well as physical capacity of the animals. Moreover, multiple pathological processes were simultaneously ameliorated following I/R in NBE‐treated mice, including reductions in inflammation, fibrosis, apoptosis, and senescence, alongside increased cardiomyocyte cell‐cycle reentry.

Cardiac remodeling after injury is driven by cardiomyocyte loss, immune‐cell infiltration, and progressive collagen deposition. While initially protective, these responses become maladaptive when excessive, particularly in aged populations (Tracy et al. 2020). Consistent with this, we found that plasma dilution reduced the numbers of infiltrating CD45^+^ inflammatory cells in the cardiac tissue and diminished the numbers of cardiac fibroblasts at Day 5 post‐injury. These findings suggest that administering NBE 24 h post‐injury might modulate the second phase of inflammatory response which peaks around Day 5–7 and drives collagen deposition and scar formation (Ong et al. 2018). Future studies on the immune cell sub‐populations and the stages of fibrosis, with detailed timelines and intra‐organ locations, will promote an even better understanding of the cardio‐protective effects of plasma dilution.

Regarding the clinical capacity, published work suggests that plasma dilution does not pathologically deplete systemic proteins, and in contrast, might calibrate these to healthy states in mice and in people (Mehdipour et al. 2020; Kim et al. 2022). In support of the broad tissue rejuvenation potential of this procedure, we show here that a single NBE improves cardiac health and performance in aged, non‐injured (sham) mice.

Mechanistically, our study identified that NBE normalized the total levels of determinants of TGF‐β signaling (SMAD2/3, TGF‐β1, vimentin, and α‐SMA), which are the central mediators of fibrosis and cardiac hypertrophy (Dobaczewski et al. 2011; Hanna and Frangogiannis 2019). This normalization was accompanied by reduced fibrosis across all myocardial layers. The presence of fibrosis in the endocardium and mid‐myocardium aligns with the typical wavefront of myocardial necrosis after LAD ligation, where the subendocardium is most vulnerable. Epicardial fibrosis, although less common in mild I/R, has been observed after LAD ligation (Pop et al. 2013; Meng et al. 2023) and is linked to epicardial fibroblast activation (Ruiz‐Villalba et al. 2015; Fang et al. 2016). Thus, a possible modulatory effect of NBE on fibroblast recruitment and ECM deposition might involve alteration in the spatial pattern of scar formation, which would be very interesting to explore in future work.

Moreover, while physiological TGF‐β signaling supports cardiomyocyte growth and ECM deposition to maintain wall integrity, excessive activation promotes apoptosis, fibrosis, and hypertrophy, leading to pathologic remodeling (Dobaczewski et al. 2011; Hanna and Frangogiannis 2019; Bujak and Frangogiannis 2007; Euler 2015). Thus, balancing inflammatory and fibrotic signals is the major goal in developing MI treatments. Here, we show that a single plasma dilution has the potential of doing so, through healthy recalibration of the levels of select protein determinants (Bujak and Frangogiannis 2007; Dobaczewski et al. 2011). Moreover, our study explored the signaling intensity of the TGF‐β pathway, and the findings suggest that NBE attenuates but does not abrogate TGF‐β signaling through diminishing the levels of phosphorylated Smad3; this might limit pathological fibrosis and hypertrophy while preserving adaptive repair mechanisms.

Beyond modulation of inflammatory and fibrotic signaling, apoptosis and senescence represent additional pathologies of post‐injury remodeling, leading to excessive cardiomyocyte loss and dysregulated fibrosis (Teringova and Tousek 2017). Our study showed that plasma dilution significantly reduced proapoptotic markers (Bax2), activated caspase 3, and reduced TUNEL positive cardiomyocytes in the infarct and border area, 5 days after I/R. Coupled with reduced p16^+^ senescent cells and augmented cell cycle re‐entry as evidenced by increased Ki67^+^ cells, these effects likely contribute to preserved LV wall thickness in I/R + NBE mice.

Molecularly, our data suggest that the benefits of NBE at 5 days post‐I/R involve downregulation of 12 proteins that are known to contribute to chronic inflammation and excessive ECM deposition, including IL‐3, IL‐5, IL‐17, LRP6, IFN‐alpha (Penna et al. 2020; Li et al. 2016; Lai et al. 2021), and were found to be mainly associated with the JAK/STAT pathway, which is compelling and warrants future functional studies, as this pathway has a similar double‐edged nature to that of TGF‐β, providing beneficial effects at physiological levels but contributing to tissue and cellular damage when excessively activated (Dobaczewski et al. 2011; Massagué and Sheppard 2023; Boengler et al. 2008). Additionally, no upregulated de‐novo synthesized proteins were found in the I/R + NBE group, as compared to I/R alone, suggesting that cardiac damage causes protein overproduction rather than underproduction, and that NBE is cardio‐protective through ameliorating the pro‐inflammatory and profibrotic protein synthesis post‐I/R.

At 3 months post I/R, differentially expressed proteins were evident in sham versus I/R groups and their identity reflected chronic cardiac remodeling post MI (Leancă et al. 2022; Arai 2015). However, no DEPs were found between I/R versus I/R plus NBE, suggesting that chronic cardiac remodeling continues in both groups. These findings suggest that modulation of the key early processes: inflammation, fibrosis, and apoptosis by an NBE at 24 h post I/R transiently shifted the trajectory of cardiac wound healing allowing a better functional recovery during the process of chronic heart tissue remodeling. This notion is consistent with previous reports demonstrating that the magnitude and timely resolution of early inflammatory and fibrotic responses after MI are key determinants of better functional recovery and lower mortality risk (Prabhu and Frangogiannis 2016; Frangogiannis 2014; Fonseca and Izar 2022). Future large studies are clearly needed to further explore the precise temporal dynamics of the molecular changes in cardiac remodeling and comprehensively evaluate the systemic effects and safety profile of NBE, including its potential to promote multi‐organ resilience following cardiac injury.

Nonetheless, these findings advance our understanding of how plasma dilution promotes cardiac repair and highlight the need for comprehensive identification of different heart‐resident cell types that might have different proliferation and senescence properties in the control, NBE, I/R, and I/*R* + NBE cohorts.

## 4 Conclusion

Our work established an important pre‐clinical model and advanced the understanding of how broad recalibration of systemic milieu can ameliorate aging‐associated cardiac pathology. It demonstrated that a single plasma dilution in aged mice performed 24 h post‐I/R is sufficient to normalize de‐novo synthesis of the key classes of proteins that are relevant for healthy cardiac remodeling. Building on these findings, future studies are expected to extend this work to its specific clinical potential and potentially, in a younger cohort.

In future work, it will be important to analyze additional timepoints after injury, different plasma dilution percentages, and roles of sub‐populations of leukocytes, fibroblasts, cardiomyocytes, and heart resident cells in cardiac pathology and NBE‐promoted cardio‐protection.

In vivo gain and loss‐of‐function studies need to be developed to expand the JAK/STAT and TGF‐β signaling determinants identified by our bioinformatics analyses and to further explore the molecular, cellular, and tissue changes after NBE. The sex‐specific differences might emerge with higher numbers of examined samples. Investigations into the overall safety profile of plasma dilution for longer than three months would be important.

Nonetheless, our study showed that plasma dilution that is performed 24 h post even mitigates key pathological processes (i.e., inflammation, fibrosis, apoptosis, senescence) post‐ I/R injury, promoting adaptive cardiac remodeling, preserving cardiac function, and improving physical performance of aged animals. These findings expand the therapeutic application of plasma dilution and underscore the promise of systemic interventions as a complementary strategy for managing MI.

## 5 Methods

### 5.1 Study Design

The study aimed to evaluate the therapeutic potential of NBE using in vivo murine models of acute myocardial ischemia–reperfusion, specifically by assessing cardiac function, motor function, and endurance and characterizing key molecular mechanisms post‐injury. Our approach models myocardial I/R injury in old C57BL/6 mice (18–22 months: 3 males and 3 females), as in Xu et al. (2014), and follows NBE procedure optimized for murine models, as described in Mehdipour et al. (2022). The use of C57BL/6 mice were used for this experiment for its reproducibility (Salto‐Tellez et al. 2004), well‐defined immunological background after MI (Mitre et al. 2023; Yan et al. 2013), and extensive prior use in experimental MI research. Furthermore, both male and female mice were used to ensure that the observed effects of the intervention were generalizable across sexes and to account for sex as a biological variable (Lindsey et al. 2021; Miller et al. 2017). All experiments involve 4 groups (Sham, Sham + NBE, I/R vehicle, I/R + NBE), where each group has randomly assigned six mice. The 5‐day and 3‐month time points were selected to determine changes in key mechanisms (i.e., inflammation, fibrosis, etc.) post‐injury and extent of infarct size, respectively. For the groups observed for 3 months, monthly data for cardiac function, motor function, and endurance were collected. For all surgeries and analysis, codes were used and scientists assessing/quantifying the results were blinded. All experiments were replicated as indicated.

All procedures were performed in accordance with the administrative panel of the Office of Laboratory Animal Care, UC Berkeley. The protocol was approved by the UC Berkeley Animal Care and Use Committee (ACUC).

### 5.2 Myocardial Ischemia–Reperfusion Injury

To model myocardial infarction, myocardial ischemia reperfusion (I/R) injury was induced in C57BL/6 mice following Xu et al. (2014) procedure with some modifications. Buprenorphine (0.1 mg/kg) was administered intraperitoneally before the surgery. The mouse was anesthetized with isoflurane in oxygen (2%–5%) at 0.5–1 L/min to full relaxation and maintained in 2% isoflurane in oxygen. A 1‐mL saline solution was subcutaneously injected in the dorsal back. The left chest was shaved to provide sufficient exposure. The mouse was fixed on the operating table by anchoring its upper jaw through tying its upper incisor to the platform. The mouse was intubated using a 1‐in G22 IV catheter attached to a ventilator set. The ventilator settings were calibrated based on the mouse's body weight with tidal volume set at 5–10 mL/kg BW at 150 beats per minute (bpm). The mouse was repositioned to right lateral decubitus and secured by taping the legs, arms, and tails to the platform. The surgical area on the left chest was scrubbed three times with betadine and injected with 0.5% lidocaine/0.25% bupivacaine (7 mg/kg) subcutaneously.

Once prepared, thoracotomy was done by making a 1‐cm oblique incision along the left sternal border to the left axillary. The fourth intercostal space was exposed and cut open by < 1 cm and the pericardium was torn to expose the heart. Upon locating the LAD, an 8‐0 prolene suture was passed approximately 2 mm lower than the tip of the left auricle and loosely double tied, leaving a 2–3 mm diameter loop where a polyethylene (PE)‐10 tubing was placed. The loop was tightened and secured by doing a slip knot. Ligation was kept for 30 min and success was assessed by the blanching and akinesis of LV downstream of ligation. After inducing ischemia, the PE tubing and prolene suture were removed. The reperfusion was assessed by checking the return of pink‐red color of LV anterior wall. Mice that did not show any blanching nor akinesis downstream of LV during ischemic induction and return of pink‐red color of LV during reperfusion were excluded from the experiment.

The ribs were closed using a 4‐0 nylon suture and the skin by wound clips. Once closed, isoflurane flow was stopped while continuing the supply of oxygen. The ventilation was removed after the mouse attempted to breathe spontaneously. During the subsequent 48‐h recovery period, the mice were closely monitored for postoperative complications. Exclusion criteria included: (1) death within the first 48 h after myocardial I/R; (2) severe pain, as indicated by inactivity, hunched posture, decreased self‐grooming, and a stiff gait; and (3) severe bleeding and/or swelling around the incision site. No mice met any of these exclusion criteria; hence, no animals were excluded from the study.

The mice randomized to sham surgery received the same surgery using the same instrumentation as the mice with I/R injury, except that the 8‐0 prolene suture was only passed around the LAD but not tied. Sham mice had the same duration of anesthesia, ventilation, and post‐operative care as the I/R group.

Each mouse was observed and sacrificed at 5 days or 90 days post‐injury.

### 5.3 Blood Exchange

Neutral blood exchange was performed in old mice 24 h after myocardial ischemia–reperfusion. Initiating the NBE treatment at 24 h was decided based on the temporal response post‐injury and the ethical limitations of doing one (1) major surgical procedure per mouse per day based on University of Californica (UC) Berkeley Animal Care and Use Committee (ACUC) guidelines. https://acuc.berkeley.edu/guidelines/Surgical%20Procedures.pdf. As this study is the first to study the effects of NBE on MI, exemptions to do 2 major procedures was not feasible.

To prepare the mice for NBE, mouse underwent jugular vein catheterization as in Mehdipour et al. (2022). Complete blood solution (exchange fluid) was freshly prepared prior to doing the plasma exchanges. Blood from anesthetized old donor mice was obtained by terminal cardiac puncture using a 3 mL hypodermic needle prefilled with 10 units of heparin. The extracted blood was centrifuged at 500 *g* for 5 min at room temperature and the platelet‐rich plasma fractions were discarded. The remaining blood cell pellets were washed with normal saline solution (NSS, 0.9% NaCl) and replaced with 5% mouse serum albumin (MSA) (Innovative Research, IMSALB100MG) in NSS at a volume equal to the discarded plasma.

For the plasma exchange, the small‐animal plasma exchange device was first calibrated to an exchange volume of 300 μL in each direction prior to starting. This translates to an effective exchange of volume of 250 μL as the tubing has 50 μL dead space. Of note, one full exchange is equal to one successful transfer of complete blood solution to recipient mouse. Mice are estimated to have ~58.5 mL of blood per kg of body weight (George et al. 2023; Parasuraman et al. 2010), which is the value of reference in determining the volume of blood to be exchanged to achieve 50% exchange.

Once prepared, blood exchange in catheterized mice was performed. Blood exchange proceeded by withdrawing blood (250 μL) from the recipient mouse then delivering the same volume of 5% MSA. This is considered one cycle of blood exchange. The cycle continues for approximately 3–4 exchanges denoting ~50% replacement of blood plasma. Once the entire procedure is completed, the catheters were removed, the vessels were tied with 4‐0 nylon suture and the skin was closed using wound clips. Mice were observed closely during the first week, monitoring their recovery. Mice with (1) severe pain: inactivity, hunched posture, decreased self‐grooming, and stiff gait, as well as (2) severe bleeding and/or swelling around the incision sites were euthanized.

### 5.4 Azido‐Homo‐Alanine Treatment

After injury, mice were injected intraperitoneally with azido‐homo‐alanine (Jena Biosciences CLK‐AA009, 0.02 mmol/kg) daily for 5 days.

### 5.5 MRI

Magnetic Resonance Imaging (MRI) was performed at baseline, 30, 60, and 90 days post‐LAD ligation on the horizontal bore 7.0 T scanner (PharmaScan 70/16 US; Bruker Biospin; Ettlingen, Germany). Mice were anesthetized using isoflurane (2%–3%) with 1 L/min oxygen and administered through a mask covering the nose and mouth of the mice. The mice were maintained at a body temperature of 36.5°C–37°C, with water flow regulated, and continuously monitored using a physiological monitoring system (SA Instruments Inc., Stony Brook, NY, USA). The respiration rate was also maintained at 30–35 breaths per minute.

Once correctly orientated, the whole heart was imaged using FLASH cine MR sequence. Each MR sequence is initiated by the R wave of the electrocardiogram (ECG), which corresponds to end diastole. Short, continuous axis slices with a 0.8 mm thickness were acquired so that the whole LV could be visualized. Images were exported to the universal Digital Imaging and Communications in Medicine (DICOM) format and analyzed using Image J. All images for each individual short axis slice were combined to give a stack image showing the heart throughout the cardiac cycle.

End‐diastolic (ED) and end‐systolic (ES) frames were selected as those with the largest and smallest cavity volumes, respectively. Epicardial and endocardial borders were outlined using the free‐hand drawing function of Image J. Left ventricle anatomical and functional parameters were calculated as described in Stuckey et al. (2008). Briefly, ED volume (EDV) and ES volume (ESV) were calculated by multiplying the cavity areas in all frames with slice thickness (0.8 mm). Stroke volume (SV) was calculated as the difference between EDV and ESV. Other parameters measured are ejection fraction (EF = SV ÷ EDV), cardiac output (CO = SV × heart rate), cardiac index (CI = CO ÷ body mass), and LV mass (LVM = (EDV − epicardial volume) × myocardial specific gravity (1.05)).

### 5.6 Treadmill Test

To evaluate endurance and exercise capacity of mice, treadmill test (TMT) was performed at baseline, 30‐, 60‐, and 90‐days post‐LAD ligation. Prior to testing, mice were trained for 2 days at low speed of 5–8 m/min for 10 min on Day 1 and 5–10 m/min for 10 min on Day 2. On the third day, mice ran at a start rate of 8 m/min, which is then increased by 1 m/min every 10 min after the 10 min mark. All mice ran until exhaustion or once they reached 180 min. Exhaustion is determined by refusal to run on the treadmill for at least 10 s. All mice were fasted for 2 h before running, with access to water ad libitum.

### 5.7 Hanging Test

To evaluate motor function of mice after MI, the hanging test was measured at baseline, 30‐, 60‐, and 90‐ days post‐LAD ligation. This test was performed using a box (26 in × 19 in × 19 in) covered with a screen box (3 in × 19 in × 19 in) with 1 cm mesh and 1 mm wire. Soft bedding was placed at the bottom of the box to serve as a cushion.

For the main test, mice were placed on the screen box. Once the mice's grip with the wires was ensured, the screen box was inverted to hang the mice at a 25 cm height. The test ended once the mice fell or once they reached 300 s. The test was repeated for 3 times with at least a 5 min rest between trials. Hanging time below 10 s was not considered unless the mouse was unable to hang on for at least 10 s during all three attempts.

### 5.8 Tissue and Serum Collection

Tissue and serum collection were performed 5 days and 90 days after injury. During these time points, blood was collected through cardiac puncture using a 1‐mL syringe with a 25‐gauge needle. Serum was collected by allowing the blood in 1.5‐mL microcentrifuge tubes to clot at room temperature for 30 min, followed by centrifugation at 1000 *g* for 5 min at 4°C and collecting the supernatant. After blood collection, the heart was intracardially perfused with cold 1× PBS and isolated. The lower third of the heart was placed in a microcentrifuge tube and stored at −80°C for further analysis. The upper two‐thirds of the heart was embedded in OCT (Sakura Finetek, 4583), snap frozen in methyl butane, cooled to −70°C in dry ice, and stored at −80°C until analysis.

### 5.9 Masson's Trichrome Staining

Masson's Trichrome staining was performed according to the manufacturer's protocol (Abcam ab150686), with some modifications. Samples collected 5 days and 90 days after injury were used. 10 μm heart sections were fixed with 4% paraformaldehyde (PFA) and preheated in Bouin's solution (60°C) for 1 h each. Then, each slide was stained with Weigert's iron hematoxylin for 7 min, Biebrich scarlet‐acid fuchsin for 27 min, phosphomolybdic‐phosphotungstic solution for 7 min, and aniline blue solution for 30 min, and rinsed in 1% acetic acid solution for 3 min. All sections were dehydrated in 70%, 95%, and 100% ethyl alcohol and cleared in xylene. 2–3 drops of xylene mounting medium were added and coverslips were placed.

For heart sections, ten different areas for infarct, border, and remote zones were obtained at 200× magnification per sample (Wu et al. 2024). For liver sections, samples collected at 3 months post I/R were used as follows: 15–20 different areas obtained from five sections per sample and all were imaged; this method is typical, as per (Arjmand et al. 2020). Images were post‐processed with ImageJ and quantified by two analysts who were blinded to sample identification. Fibrotic indices were quantified by dividing total fibrotic area (mm^2^) by total tissue area (mm^2^) per image (Figure S3).

### 5.10 Oil Red O Staining

Oil Red O staining was performed according to the manufacturer's protocol (Sigma OO625‐25G), with some modifications. Samples collected 3 months after injury were used. 10 μm heart sections were fixed with 4% paraformaldehyde (PFA) for 10 min then submerged in 60% isopropanol for 2 min. Then, each slide was stained with Oil Red O working solution (24 mL of a 0.5% stock solution dissolved in 16 mL distilled water) for 15 min, washed with water, counterstained with Mayer's modified hematoxylin for 2 min, and washed with water for 1 min. All slides were mounted using 9:1 glycerol:PBS and sealed with nail polish. Thirty random images were obtained per mice from five sections and post‐processed with ImageJ. Two analysts blinded to sample identification performed the quantification. Percent lipid area was quantified by dividing total lipid area (mm^2^) by total tissue area (mm^2^) per image (Figure S4).

### 5.11 Immunofluorescence Staining

Immunostaining on fresh frozen cardiac tissue obtained 5 days after injury was performed. 10 μm heart sections were fixed with 4% PFA for 10 min, permeabilized with 0.2% Triton X‐100 + 1% bovine serum albumin (BSA) in PBS, blocked with 10% serum in PBS for 1 h. Permeabilization was skipped for membrane‐associated proteins. Slides were incubated with desired primary antibodies (Table S1) at 4°C overnight. The following day, slides were washed with a wash buffer and incubated with secondary fluorophore‐tagged antibodies. The slides were mounted with 2–3 drops of fluoromount‐G with DAPI (Invitrogen, 501128966) and viewed under a fluorescent microscope. IgG controls with isotype‐matched antibodies were routinely done, and non‐specific fluorescence was minimal.

Ten random representative areas for infarct, border, and remote zones were imaged at high magnification (200×) per sample. Percent positive cells were quantified by dividing positive cells by total number of nuclei per image × 100. These analyses were performed and quantified using ImageJ by two analysts blinded to sample identification.

### 5.12 TUNEL Assay

Cell death was evaluated with an in situ Cell Death Detection Kit (Invitrogen C10617) for TUNEL staining as directed by the manufacturer's instructions with some modifications as frozen tissue sections were used in the study. The sections were co‐stained with vimentin (CST 5741S, 1:500) or alpha actinin (Invitrogen MA106100, 1:500) to identify the percentage of TUNEL+ cardiac fibroblast and TUNEL+ cardiomyocytes. Ten random representative areas for infarct, border, and remote zones were imaged at high magnification (200×) per sample. The percentage of TUNEL+ cells was quantified by dividing positive cells by the total number of nuclei per image × 100. These analyses were performed and quantified using ImageJ by two analysts blinded to sample identification.

### 5.13 Wheat Germ Agglutinin (WGA) Staining

To assess cardiomyocyte hypertrophy, sections were stained with wheat germ agglutinin (WGA) (Invitrogen W11262, AF594 conjugate) following the manufacturer's protocol. CM hypertrophy was assessed by the cross‐sectional area of cardiomyocytes (150 cells per mouse) labeled with WGA around the border and remote zone. To control for tissue orientation, only cardiomyocytes that were surrounded by capillaries and displaying a cross‐sectional orientation were analyzed. Cross‐sectional area was quantified using ImageJ by two analysts blinded to sample information.

### 5.14 Western Blot

Protein lysates were separated via gel electrophoresis, transferred to a polyvinylidene fluoride membrane, and blocked with 5% non‐fat dry milk for 1 h. The membranes were incubated overnight with primary antibodies (Table S2) at 4°C, washed, incubated with horseradish peroxidase (HRP)‐conjugated secondary antibody at room temperature for 1 h, exposed via chemiluminescence with ECL (Advansta K‐12045‐D50), and visualized with Analytik Jena UVP ChemStudio imager. Full‐length raw blots are shown in Figures S10 and S11.

### 5.15 Antibody Array Proteomics

Levels of 308 proteins (L‐308 glass array, RayBiotech, Norcross, GA, USA) were measured in heart five days post‐injury following manufacturer's protocol. Briefly, click chemistry (Thermo Fisher Scientific C10276) was performed to biotinylate (Thermo Fisher Scientific B10185) AHA‐tagged proteins as directed by the manufacturer's instructions. Click western blot was performed to confirm biotinylation. Clicked protein lysates were dialyzed in 0.2% Triton X‐100 in PBS. Meanwhile, the array membranes were incubated with blocking buffer overnight at 4°C, then with 30 μg of clicked‐dialyzed heart overnight at 4°C. The arrays were washed, incubated with Streptavidin‐conjugated Cy3 fluorescence dye overnight at 4°C, washed, dried, and scanned using GenePix Pro 6.1 software (Molecular Devices). Fluorescence intensities were obtained for each array. Median spot intensities were extracted in the R language (R Core Team 2021) with limma (Ritchie et al. 2015). Background correction was performed with the normexp‐by‐control method (Shi et al. 2010), utilizing positive and negative control spots, and an offset of 20. The values were normalized with the positive control spot (POS) mean signal intensity as suggested by the array L308 manual (https://doc.raybiotech.com/pdf/Manual/AAM‐BLG‐1.pdf), where values are scaled by a factor from the ratio of the mean POS spot signal on an arbitrary reference array to the mean POS spot signal on all other arrays. Differential expression analysis was conducted by averaging each probe replicate pair, then using the limma‐trend method with a group means parameterization design and contrast matrix, and running parametric ebayes(). DEPs were identified with the decideTests(), applying a log2 fold change threshold of 1 and FDR‐controlled *p*‐value threshold of 0.05. Overrepresentation analysis and visualization were performed by manually mapping each protein to its corresponding gene and entrez‐ids in the mouse genome informatics database (Baldarelli et al. 2024). Protein–protein interaction networks were constructed using STRING's web API (Szklarczyk et al. 2025). All other visualizations were produced with ggplot2 R package (Wickham 2009).

### 5.16 Statistics

Sample sizes were determined by a power analysis to reach at least 85%, noting an expected treatment effect difference of 10% with a standard deviation of 5% (variance 25%) and a significance threshold of α < 0.05 (Lindsey et al. 2018, 2021). The mice were randomized (*n* = 6 per group, 3 male and 3 female) and blinded for analysis. All data analyses were either performed using Prism v.8 software from GraphPad, Python, and Microsoft Excel 2019. Raw data per figure can be found in Data S2. Unpaired *t*‐tests (two‐sided) were applied to comparisons between two groups if variances were significantly different between groups, with statistical significance defined as *p* < 0.05. For comparisons among more than two groups, one‐way analysis of variance (ANOVA) was used, followed by Dunnett's post hoc test for adjusted *p* values (significant at an adjusted *p* value threshold < 0.05). When normality was not satisfied, Mann–Whitney tests were performed on comparisons among groups/conditions.

## Author Contributions

J.M.C.C. contributed to the planning of the study, performed the surgeries and the experiments that are shown in all figures, analyzed and interpreted the data, and wrote the manuscript. R.A. performed Trichrome staining, antibody arrays of 5‐day heart samples, vimentin, Ki67, WGA, and TUNEL immunostaining of Figures 2, 3, 4, 5, 6, and assisted with surgeries. H.Y. contributed to the functional assays in Figure 1 and assisted with surgeries. Z.M. contributed to the TUNEL assay and vimentin staining of Figures 1, 3, and 4 and to the analyses of MRI images. N.M. performed p16 immunofluorescence of Figures 4 and 5 and assisted with data analyses. Q.Y. performed the proteomic analysis of Figures 6B–G and S2 and S7. Z.R.R. performed antibody arrays for 3‐month heart samples in Figure S7. Z.R.R. and K.R.M. performed the liver studies and provided Figure S2. M.J.C., A.R.M., and J.B.N. contributed to the planning, data interpretation, and manuscript writing. I.M.C. planned, directed, integrated, and interpreted the work and wrote the manuscript.

## Funding

This work was supported by Department of Science and Technology‐PCHRD REP, Commission on Higher Education PCARI IHITM (UC Berkeley PCARI‐CHED) 2018–033 collaborative grant to I.M.C. and J.M.C.C., National Institute on Aging (NIA) grant NIA T32 AG000266 to ZR, National Institutes of Health (NIH) grant NHLB R01 139605 to I.M.C., NIA grant R01AG071787 to I.M.C., Open Philanthropy award to I.M.C., CDMRP/USAMRDC TX230133 grant to I.M.C.

## Conflicts of Interest

Irina Conboy is founder and CSO of Generation Lab.

## Supporting information


**Data S1:** BONCAT array data.

**Data S2:** Raw Data Per Figure_v2.

**Figure S1:** Survival curve analysis of mice subjected to three ischemia durations (30, 40, and 60 min), with or without NBE, showed reduced survival in older mice (20–22 weeks) exposed to 40‐ and 60‐min ischemia. (A) Schematic diagram of the initial experiment. Neutral blood exchange (NBE) was performed 24 h after myocardial ischemia–reperfusion (I/R) injury. Mice were observed for 30 days. (B) Kaplan–Meier survival curve. (*n* = 4 per group, *p* < 0.05). Of note, old mice administered with NBE at 24 h post 40‐min I/R tended to survive better than I/R alone group.
**Figure S2:** NBE does not promote hepatic inflammation, fibrosis, or lipid accumulation. (A) Representative Masson's' trichrome images 3 months after injury per treatment showed no changes on collagen deposition. (B) Representative Oil Red O images 3 months after injury per treatment showed no changes on lipid accumulation. (C, D) Percent fibrotic and lipid area across all groups, respectively, revealed no significant changes on hepatic fibrosis and lipid accumulation. (E) Volcano plots revealed IL‐23 to be upregulated in liver of I/R + NBE compared to I/R group 3 months post‐I/R. Protein highlighted in red is significantly upregulated (adjusted *p*‐value < 0.05 (horizontal dotted line) and log2 fold change > 2 (vertical dotted line)). Scale: 100 μm. Data are shown as the mean ± SE (*n* = 6; 3 males, 3 females). ns = no significance.
**Figure S3:** The approach for quantifying liver fibrosis. Liver sections were processed and stained with Masson's trichrome. Images were obtained from five different sections and blue‐stained collagen fibers and total tissue area were analyzed using the deconvolution function on Image J. (A) Expanded “Image” menu, showing selection of “Color threshold” under the “Adjust” setting. (B) Manual thresholds of the Hue (135–215), Saturation (0–255), and Brightness (0–255) were set as the values to select the optimal blue color intensity. This threshold selects the collagen area as outlined in yellow in the “selected area” image. (C) Manual thresholds of the Hue (100–255), Saturation (0–255), and Brightness (0–255) were set as the values to select the total tissue area. This threshold selects tissue area with positive nucleus and cytoplasm as confirmed by the yellow outline shown in the “selected area” image.
**Figure S4:** The approach for quantifying liver adiposity. Liver sections were processed and stained with Oil Red O. Images were acquired from five different sections and lipid and total tissue area were analyzed using the deconvolution function on Image J. (A) Expanded “Image” menu, showing selection of “Color deconvolution” under the “Color” setting. (B) Deconvoluted images showing several channels (color1: R: 0.72, G: 0.99, B: 0.11; color2: R: 0.65, G: 0.70, B: 0.29; color3: R: 0.27, G: 9.57, B: 0.78). (C) Manual threshold of 0–200 was set to select the extracted lipid area from generated color1 image. Under the “Analyze” menu, particle size of at least 25 pixels with circularity of 0–1 were defined as positive lipid area. (D) Manual threshold of 0–200 was set to select the extracted tissue area from generated color2 image. This threshold selects tissue area with positive nucleus and cytoplasm as confirmed by the yellow outline shown in the “selected area” image.
**Figure S5:** NBE improves cardiac function and physical performance after ischemia–reperfusion in male and females. (A–C) Cine‐MRI measurements of cardiac morphology and function every month for 3 months post‐I/R revealed preserved EDV, ESV, and ejection fraction (EF) in I/R + NBE group. (D, E) Hanging indices and running distance over time for 3 months and percent (%) change from baseline showed preserved motor function and endurance in I/R + NBE group. Data are shown as the mean ± SE (*n* = 3 males, 3 females).
**Figure S6:** NBE alleviates cardiac remodeling after ischemia–reperfusion in male and females. (A) Wall thickness (in mm) in the infarct area 3 months after injury were preserved in I/R + NBE mice. (B, C) Percent infarct area and viable area. (D) Quantification of WGA staining for cardiomyocyte area revealed no significant changes on hypertrophy in I/R + NBE group. All counts were made in border zone and remote zone. Data are shown as the mean ± SE (*n* = 3 males, 3 females). ns = not significant
**Figure S7:** NBE reduces inflammation and fibrosis after ischemia–reperfusion in males and females. (A) Percentage of CD45‐positive cells in the left ventricle of samples across all groups. (B) Percentage of Vimentin‐positive cells in the left ventricle of samples across all groups. All counts were made in infarct zone, border zone, and remote zone. (C) Western blot analysis of fibrosis markers such as pSMAD2/3, SMAD2/3, pro‐TGFβ1, TGFβ1, Vimentin, α‐smooth muscle actin (SMA), and vinculin confirmed reduced fibrosis in I/R + NBE group. Data are shown as the mean ± SE (*n* = 3 males, 3 females). ns = not significant.
**Figure S8:** NBE attenuates apoptosis and senescence and promotes cell proliferation after ischemia–reperfusion in males and female. (A) TUNEL‐positive cells and (B) TUNEL+ cardiomyocytes in the left ventricle of samples across all groups. All counts were made in infarct zone, border zone, and remote zone. (C) Western blot analysis of apoptotic markers such as procaspase 3, cleaved caspase 3, and Bax2 showed reduced expression in I/R + NBE group. (D) Quantification of p16 immunofluorescent images obtained from all groups at day 5 after injury or sham operation revealed reduced apoptotic cells in I/R + NBE group. (E) Percent Ki67‐positive cells and (F) Ki67‐positive cardiomyocytes in the left ventricle of samples across all groups. All counts were made in infarct zone, border zone, and remote zone. Data are shown as the mean ± SE (*n* = 3 males, 3 females). ns = not significant.
**Figure S9:** Proteomic analysis of heart samples collected 3 months post‐ischemia/reperfusion (I/R) injury revealed distinct set of differentially expressed proteins (DEPs). (A) Schematic diagram. NBE was performed 24 h post‐I/R and heart samples were collected at 3 months post‐injury. (B) Principal component analysis (PCA) plots of the protein expression profile of all treatment groups at 3 months post‐injury revealed the clustering of all samples. Confidence ellipses at 50%, 80%, and 95% intervals are overlaid to indicate group variance. (C) Volcano plots identified the DEPs in I/R versus Sham controls. No DEPs were identified in I/R + NBE versus I/R controls. (D) Venn diagram revealed the overlapping DEPs between the upregulated proteins and their downregulation after NBE treatment 5 days post‐I/R as well as the DEPs identified 3 months post‐I/R. (E, F) Gene Ontology overrepresentation analysis of the 6 DEPs 3 months post‐I/R highlighted top 10 biological processes and molecular function, respectively. (G) cirFunMap visualization of KEGG enriched pathways of the 6 DEPs.
**Figure S10:** Unprocessed western blot detecting fibrosis markers such as pSMAD2/3, SMAD2/3, pro‐TGFβ1, TGFβ1, Vimentin, α‐smooth muscle actin (SMA), and vinculin.
**Figure S11:** Unprocessed western blot immunoblots detecting apoptotic markers such as procaspase 3, cleaved caspase 3, and Bax2.
**Table S1:** List of antibodies used for IF.
**Table S2:** List of antibodies used for WB.

## Data Availability

All data associated with this study are present in the paper or the Supporting Information.
