# Supplementary material for: Plasma Dilution After Myocardial Ischemia–Reperfusion Injury Promotes Cardiac Repair, Heart Performance, and Recovery of Motor Function and Endurance in Old Mice
Source: Aging Cell. 2026 Apr 30;25(5):e70525. doi: 10.1111/acel.70525 (PMC13130355; doi:10.1111/acel.70525)
Supplement: Supplementary file 3 — Figure S1: Survival curve analysis of mice subjected to three ischemia durations (30, 40, and 60 min), with or without NBE, showed reduced survival in older mice (20–22 weeks) exposed to 40‐ and 60‐min ischemia. (A) Schematic diagram of the initial experiment. Neutral blood exchange (NBE) was performed 24 h after myocardial ischemia–reperfusion (I/R) injury. Mice were observed for 30 days. (B) Kaplan–Meier survival curve. (n = 4 per group, p < 0.05). Of note, old mice administered with NBE at 24 h post 40‐min I/R tended to survive better than I/R alone group. Figure S2: NBE does not promote hepatic inflammation, fibrosis, or lipid accumulation. (A) Representative Masson's' trichrome images 3 months after injury per treatment showed no changes on collagen deposition. (B) Representative Oil Red O images 3 months after injury per treatment showed no changes on lipid accumulation. (C, D) Percent fibrotic and lipid area across all groups, respectively, revealed no significant changes on hepatic fibrosis and lipid accumulation. (E) Volcano plots revealed IL‐23 to be upregulated in liver of I/R + NBE compared to I/R group 3 months post‐I/R. Protein highlighted in red is significantly upregulated (adjusted p‐value < 0.05 (horizontal dotted line) and log2 fold change > 2 (vertical dotted line)). Scale: 100 μm. Data are shown as the mean ± SE (n = 6; 3 males, 3 females). ns = no significance. Figure S3: The approach for quantifying liver fibrosis. Liver sections were processed and stained with Masson's trichrome. Images were obtained from five different sections and blue‐stained collagen fibers and total tissue area were analyzed using the deconvolution function on Image J. (A) Expanded “Image” menu, showing selection of “Color threshold” under the “Adjust” setting. (B) Manual thresholds of the Hue (135–215), Saturation (0–255), and Brightness (0–255) were set as the values to select the optimal blue color intensity. This threshold selects the collagen area as outlined in yel [file ACEL-25-e70525-s001.docx]

## Supplementary Information

**Plasma dilution after myocardial ischemia-reperfusion injury promotes cardiac repair, heart performance, and recovery of motor function and endurance in old mice**

*Joana Marie C. Cruz^1,2^, Rana Alzalzalee^1^, Hayden Yeung^1^, Zainab Mahmood^1^, Qile Yang^1^, Negar Morshedian^1^,* *Zachery R. Robinson^1^, Karan R. Malhotra^1^, Michael J. Conboy^1^, Ahmad Reza Mazahery^3^, Jose B. Nevado^2,4^, Irina M. Conboy^1^**


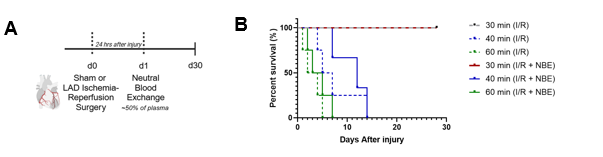


**Supplementary Figure 1. Survival curve analysis of mice subjected to three ischemia durations (30, 40, and 60 minutes), with or without NBE, showed reduced survival in older mice (20–22 weeks) exposed to 40- and 60-minute ischemia. (A).** Schematic diagram of the initial experiment. Neutral blood exchange (NBE) was performed 24 hours after myocardial ischemia-reperfusion (I/R) injury. Mice were observed for 30 days. **(B).** Kaplan-Meier survival curve. (n=4 per group, *p < 0.05*). Of note, old mice administered with NBE at 24 hours post 40-min I/R tended to survive better than I/R alone group.


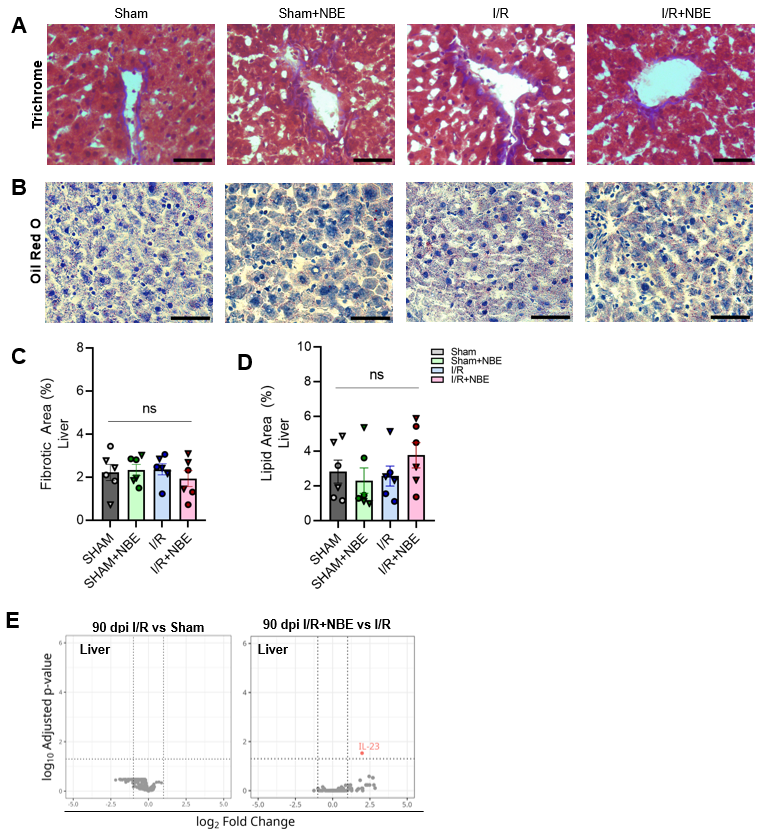


**Supplementary Figure 2. NBE does not promote hepatic inflammation, fibrosis, or lipid accumulation. (A)** Representative Masson’s’ trichrome images 3 months after injury per treatment showed no changes on collagen deposition. **(B).** Representative Oil Red O images 3 months after injury per treatment showed no changes on lipid accumulation. **(C, D).** Percent fibrotic and lipid area across all groups, respectively, revealed no significant changes on hepatic fibrosis and lipid accumulation. **(E).** Volcano plots revealed IL-23 to be upregulated in liver of I/R+NBE compared to I/R group 3 months post-I/R. Protein highlighted in red is significantly upregulated  (adjusted p-value < 0.05 (horizontal dotted line) and log2 fold change > 2 (vertical dotted line)). Scale: 100 µm. Data are shown as the mean ± SE (n=6; 3 males, 3 females). ns = no significance.


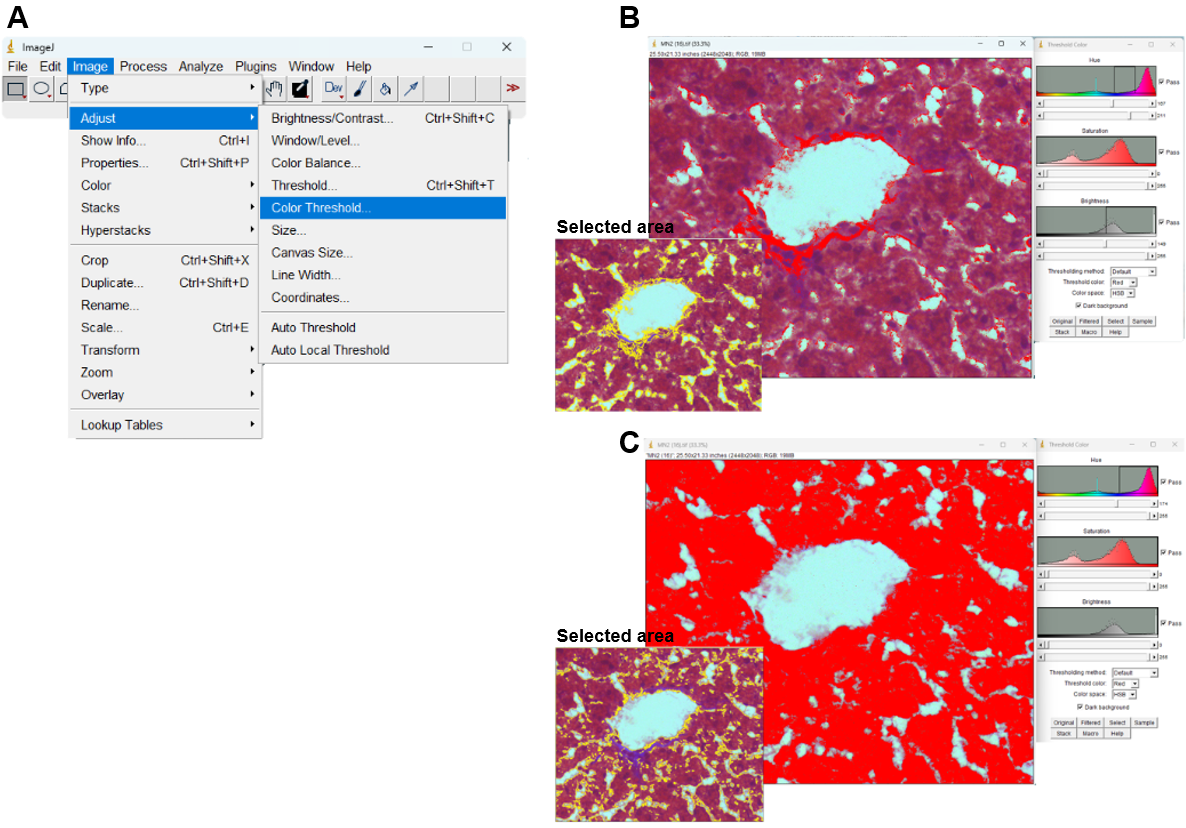


**Supplementary Figure 3.** **The approach for quantifying liver fibrosis.** Liver sections were processed and stained with Masson’s trichrome. Images were obtained from five different sections and blue-stained collagen fibers and total tissue area were analyzed using the deconvolution function on Image J. **(A).** Expanded “Image” menu, showing selection of “Color threshold” under the “Adjust” setting. **(B).** Manual thresholds of the Hue (135-215), Saturation (0-255), and Brightness (0-255) were set as the values to select the optimal blue color intensity. This threshold selects the collagen area as outlined in yellow in the “selected area” image. **(C).** Manual thresholds of the Hue (100-255), Saturation (0-255), and Brightness (0-255) were set as the values to select the total tissue area. This threshold selects tissue area with positive nucleus and cytoplasm as confirmed by the yellow outline shown in the “selected area” image.


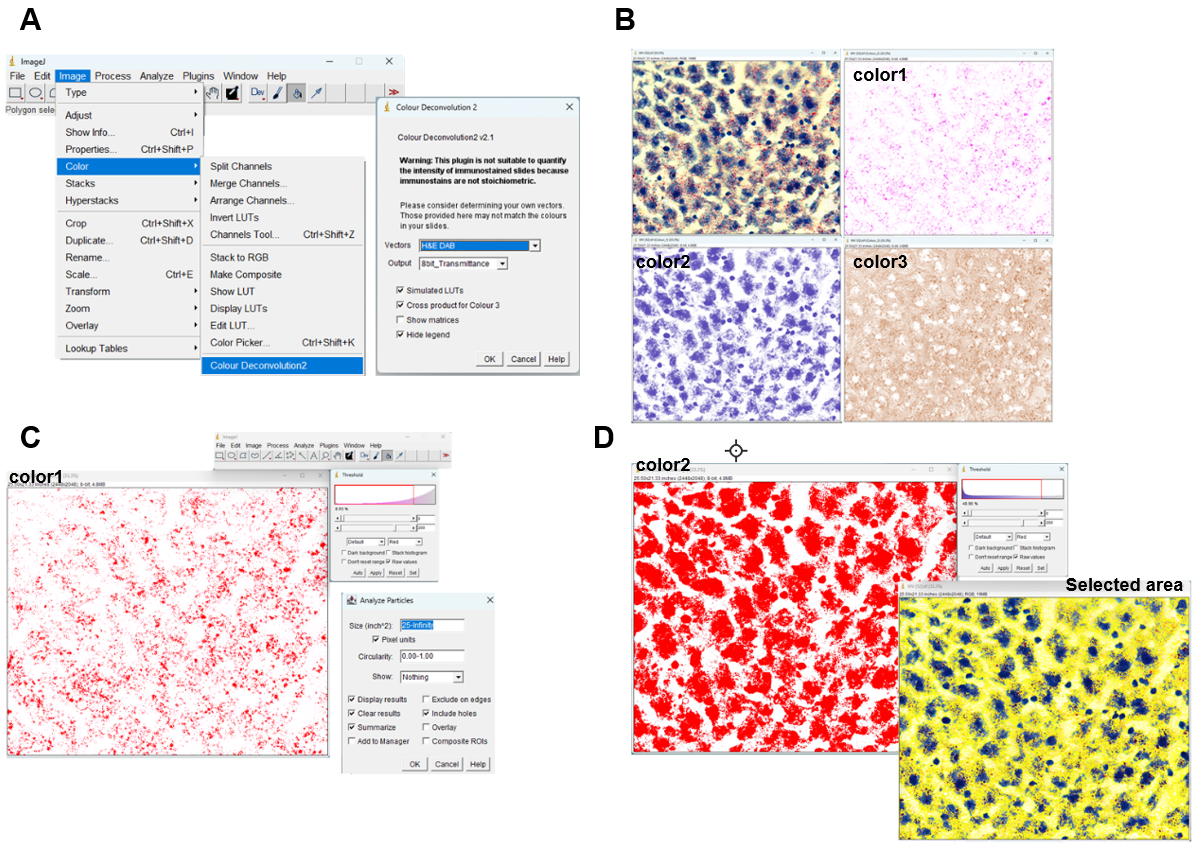


**Supplementary Figure 4**. **The approach for quantifying liver adiposity.** Liver sections were processed and stained with Oil Red O. Images were acquired from five different sections and lipid and total tissue area were analyzed using the deconvolution function on Image J. **(A).** Expanded “Image” menu, showing selection of “Color deconvolution” under the “Color” setting. **(B).** Deconvoluted images showing several channels (color1: R: 0.72, G: 0.99, B: 0.11; color2: R: 0.65, G: 0.70, B: 0.29; color3: R: 0.27, G: 9.57, B: 0.78). **(C).** Manual threshold of 0-200 was set to select the extracted lipid area from generated color1 image. Under the “Analyze” menu, particle size of at least 25 pixels with circularity of 0-1 were defined as positive lipid area. **(D).** Manual threshold of 0-200 was set to select the extracted tissue area from generated color2 image. This threshold selects tissue area with positive nucleus and cytoplasm as confirmed by the yellow outline shown in the “selected area” image.


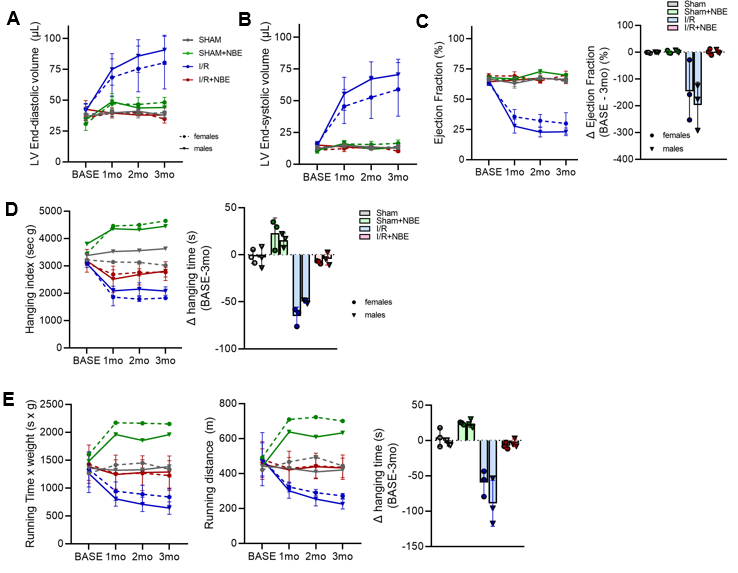


**Supplementary Figure 5. NBE improves cardiac function and physical performance after ischemia-reperfusion in male and females. (A-C)**. Cine-MRI measurements of cardiac morphology and function every month for 3 months post-I/R revealed preserved EDV, ESV, and ejection fraction (EF) in I/R+NBE group. **(D, E).** Hanging indices and running distance over time for 3 months and percent (%) change from baseline showed preserved motor function and endurance in I/R+NBE group. Data are shown as the mean ± SE (n=3 males, 3 females).


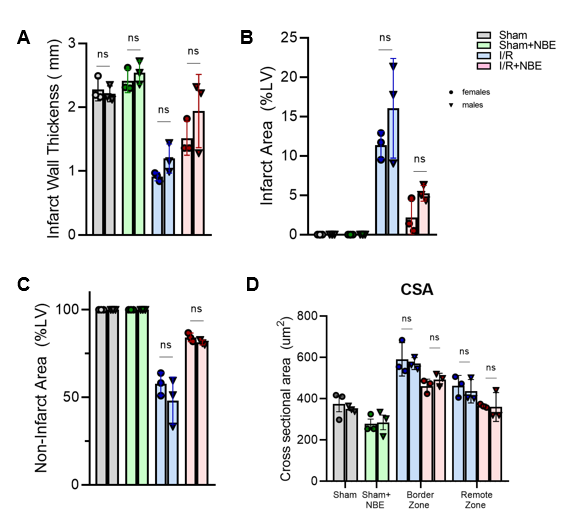


**Supplementary Figure 6. NBE alleviates cardiac remodeling after ischemia-reperfusion in male and females. (A).** Wall thickness (in mm) in the infarct area 3 months after injury were preserved in I/R+NBE mice. **(B, C).** Percent infarct area and viable area. **(D).** Quantification of WGA staining for cardiomyocyte area revealed no significant changes on hypertrophy in I/R+NBE group. All counts were made in border zone and remote zone. Data are shown as the mean ± SE (n=3 males, 3 females). ns = not significant


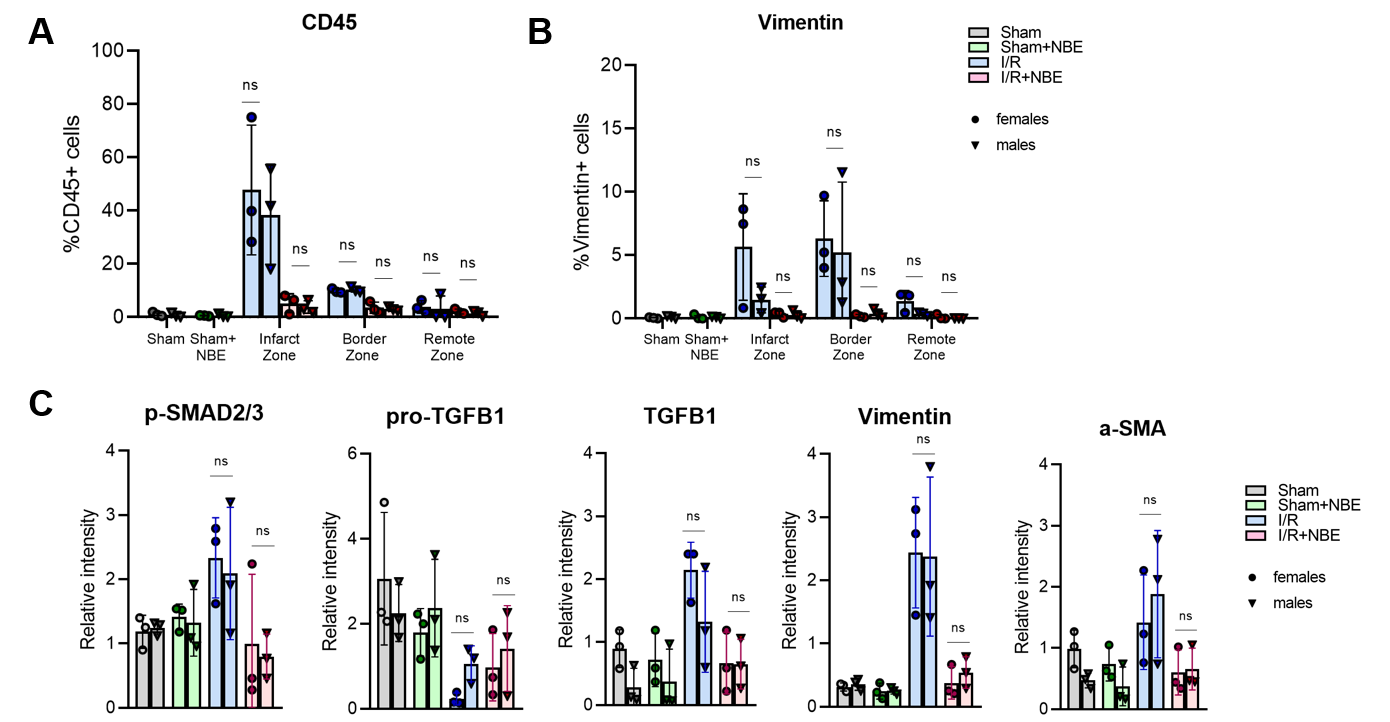


**Supplementary Figure 7. NBE reduces inflammation and fibrosis after ischemia-reperfusion in males and females. (A).** Percentage of CD45-positive cells in the left ventricle of samples across all groups. **(B).** Percentage of Vimentin-positive cells in the left ventricle of samples across all groups. All counts were made in infarct zone, border zone, and remote zone. **(C).** Western blot analysis of fibrosis markers such as pSMAD2/3, SMAD2/3, pro-TGFβ1, TGFβ1, Vimentin, α-smooth muscle actin (SMA), and vinculin confirmed reduced fibrosis in I/R+NBE group. Data are shown as the mean ± SE (n=3 males, 3 females). ns = not significant


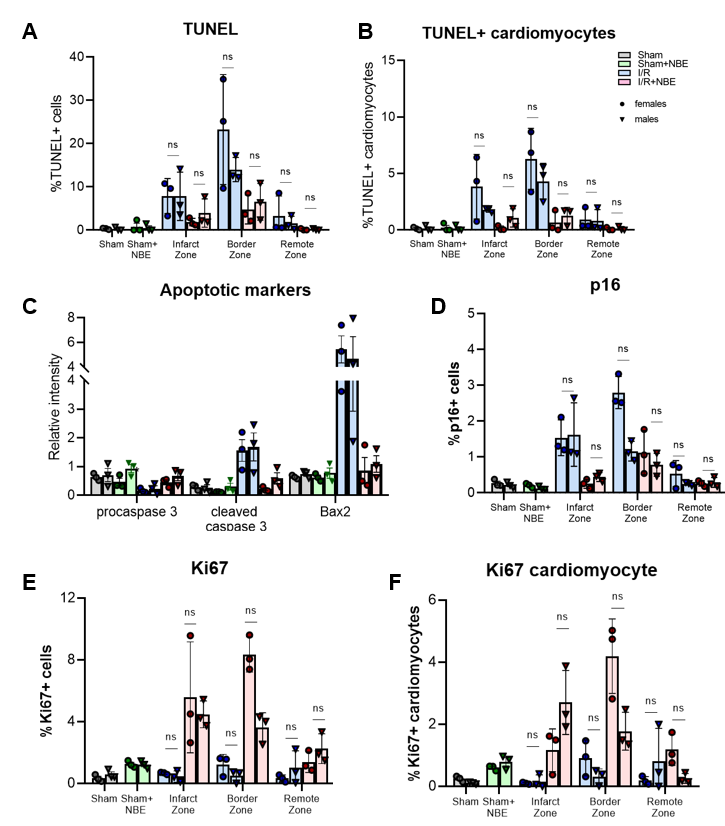


**Supplementary Figure 8. NBE attenuates apoptosis and senescence and promotes cell proliferation after ischemia-reperfusion in males and female. (A).** TUNEL-positive cells and **(B).** TUNEL+ cardiomyocytes in the left ventricle of samples across all groups. All counts were made in infarct zone, border zone, and remote zone. **(C).** Western blot analysis of apoptotic markers such as procaspase 3, cleaved caspase 3, and Bax2 showed reduced expression in I/R+NBE group. **(D).** Quantification of p16 immunofluorescent images obtained from all groups at day 5 after injury or sham operation revealed reduced apoptotic cells in I/R+NBE group. (**E).** Percent Ki67-positive cells and **(F).** Ki67-positive cardiomyocytes in the left ventricle of samples across all groups. All counts were made in infarct zone, border zone, and remote zone. Data are shown as the mean ± SE (n=3 males, 3 females). ns = not significant


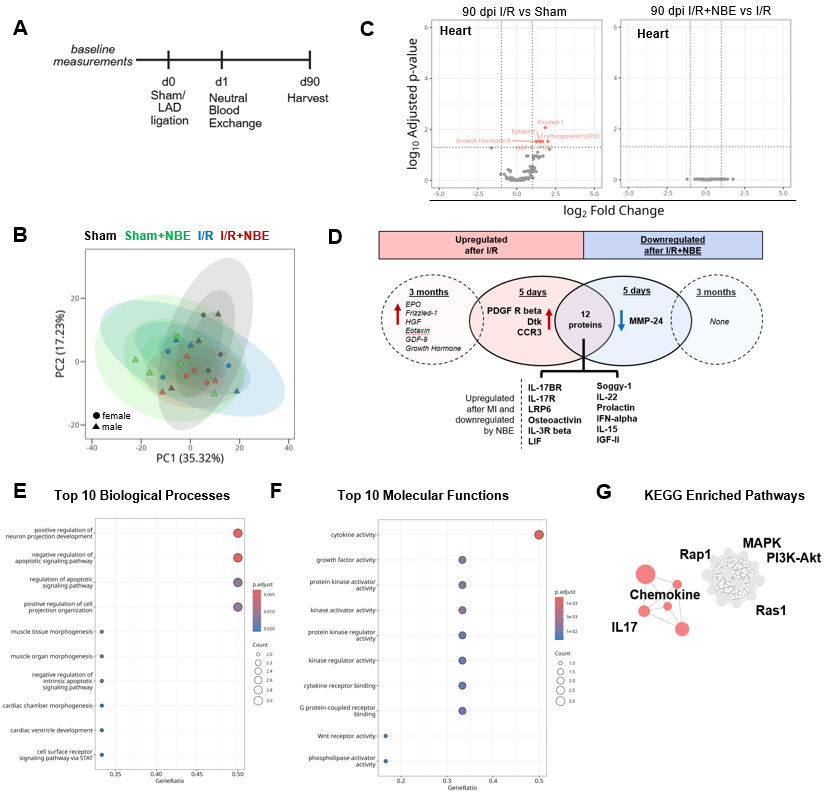


**Supplementary Figure 9. Proteomic analysis of heart samples collected 3 months post-ischemia/reperfusion (I/R) injury revealed distinct set of differentially expressed proteins (DEPs). (A).** Schematic diagram. NBE was performed 24 hours post-I/R and heart samples were collected at 3 months post-injury. **(B).** Principal component analysis (PCA) plots of the protein expression profile of all treatment groups at 3 months post-injury revealed the clustering of all samples. Confidence ellipses at 50%, 80%, and 95% intervals are overlaid to indicate group variance. **(C).** Volcano plots identified the DEPs in I/R vs Sham controls. No DEPs were identified in I/R+NBE vs. I/R controls. **(D).** Venn diagram revealed the overlapping DEPs between the upregulated proteins and their downregulation after NBE treatment 5 days post-I/R as well as the DEPs identified 3 months post-I/R. **(E, F).** Gene Ontology overrepresentation analysis of the 6 DEPs 3 months post-I/R highlighted top 10 biological processes and molecular function, respectively. **(G).** cirFunMap visualization of KEGG enriched pathways of the 6 DEPs.


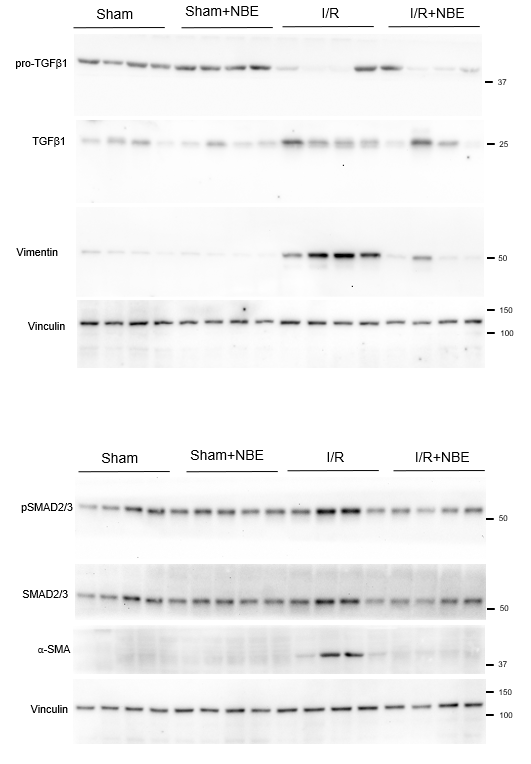


**Supplementary Figure 10.** Unprocessed western blot detecting fibrosis markers such as pSMAD2/3, SMAD2/3, pro-TGFβ1, TGFβ1, Vimentin, α-smooth muscle actin (SMA), and vinculin.


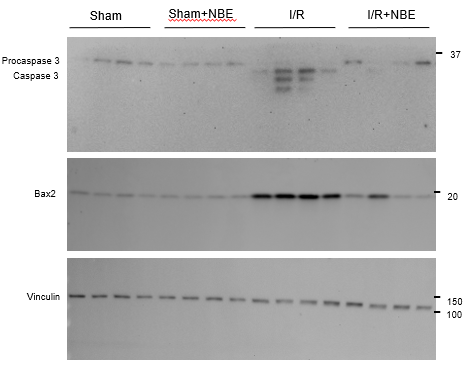


**Supplementary Figure 11.** Unprocessed western blot immunoblots detecting apoptotic markers such as procaspase 3, cleaved caspase 3, and Bax2.

**Supplementary Table 1.** List of antibodies used for IF

| Primary Antibody | | Supplier/ Code | Concentration |
| --- | --- | --- | --- |
| CD45 | Rat | CST 55307 | 1:50 |
| p16 | Rabbit | Abcam ab211542 | 1:100 |
| KI67 | Rat | Invitrogen 14-5698-82 | 1:50 |
| Vimentin | Rabbit | CST 5741S | 1:500 |
| Cardiac Troponin | Rabbit | Protein tech 15513-1-AP | 1:500 |
| Alpha-actinin | Rabbit | Invitrogen PA5-27863 | 1:500 |
| Laminin | Rat | Invitrogen MA106100 | 1:500 |
| IgG | Rat | Invitrogen PI31933 |  |
| IgG | Rabbit | CST 3900 |  |
| Secondary Antibody | | Supplier/ Code | Concentration |
| AF488 | Rat | Invitrogen A-21470 | 1:1000 |
| AF488 | Rabbit | Invitrogen A-21206 | 1:1000 |
| AF594 | Rat | Invitrogen A-11007 | 1:1000 |
| AF594 | Rabbit | Invitrogen A-11012 | 1:1000 |

**Supplementary Table 2.** List of antibodies used for WB

| Primary Antibody | | Supplier/ Code | Concentration |
| --- | --- | --- | --- |
| pSMAD 2/3 | Rabbit | Abcam 254407 | 1:500 |
| SMAD 2/3 | Rabbit | Abcam 217553 | 1:1000 |
| Vimentin | Rabbit | CST 5741S | 1:500 |
| Alpha-SMA | Rabbit | CST 19245S | 1:500 |
| TGFB1 | Mouse | Invitrogen MA1-21595 | 1:500 |
| Bax | Rabbit | CST 2772 | 1:500 |
| Pro-caspase | Rabbit | Abcam ab 184787 | 1:2000 |
| Cleaved caspase | Rabbit | Abcam ab 214430 | 1:2000 |
| VEGFA | Rabbit | Abcam ab 46154 | 1:500 |
| Vinculin | Rabbit | CST 4650 | 1:1000 |
